# A Visual and Comprehensive Review on COVID-19-Associated Pulmonary Aspergillosis (CAPA)

**DOI:** 10.3390/jof7121067

**Published:** 2021-12-11

**Authors:** Simon Feys, Maria Panagiota Almyroudi, Reinout Braspenning, Katrien Lagrou, Isabel Spriet, George Dimopoulos, Joost Wauters

**Affiliations:** 1 Medical Intensive Care Unit, University Hospitals Leuven, 3000 Leuven, Belgium; reinout.braspenning@uzleuven.be; 2 Department of Microbiology, Immunology and Transplantation, KU Leuven, 3000 Leuven, Belgium; katrien.lagrou@uzleuven.be; 3 Department of Emergency Medicine, Attikon University Hospital, National and Kapodistrian University of Athens, 12462 Athens, Greece; mariotaalm@yahoo.gr; 4 Department of Laboratory Medicine and National Reference Center for Mycosis, University Hospitals Leuven, 3000 Leuven, Belgium; 5 Pharmacy Department, University Hospitals Leuven, 3000 Leuven, Belgium; isabel.spriet@uzleuven.be; 6 Department of Pharmaceutical and Pharmacological Sciences, KU Leuven, 3000 Leuven, Belgium; 7 ICU of 1st Department of Critical Care, Sotiria Hospital, National and Kapodistrian University of Athens, 11527 Athens, Greece; gdimop@med.uoa.gr

**Keywords:** COVID-19, influenza, aspergillosis, COVID-19-associated pulmonary aspergillosis (CAPA), influenza-associated pulmonary aspergillosis, IAPA, critical care, intensive care unit

## Abstract

Coronavirus disease 19 (COVID-19)-associated pulmonary aspergillosis (CAPA) is a severe fungal infection complicating critically ill COVID-19 patients. Numerous retrospective and prospective studies have been performed to get a better grasp on this lethal co-infection. We performed a qualitative review and summarized data from 48 studies in which 7047 patients had been included, of whom 820 had CAPA. The pooled incidence of proven, probable or putative CAPA was 15.1% among 2953 ICU-admitted COVID-19 patients included in 18 prospective studies. Incidences showed great variability due to multiple factors such as discrepancies in the rate and depth of the fungal work-up. The pathophysiology and risk factors for CAPA are ill-defined, but therapy with corticosteroids and anti-interleukin-6 therapy potentially confer the biggest risk. Sampling for mycological work-up using bronchoscopy is the cornerstone for diagnosis, as imaging is often aspecific. CAPA is associated with an increased mortality, but we do not have conclusive data whether therapy contributes to an increased survival in these patients. We conclude our review with a comparison between influenza-associated pulmonary aspergillosis (IAPA) and CAPA.

## 1. Introduction

Critically ill patients with severe acute respiratory syndrome virus 2 (SARS-CoV2) are susceptible to secondary infections, including invasive pulmonary aspergillosis (IPA) caused by *Aspergillus* species. IPA is mainly described in immunocompromised patients, and specifically in severe and prolonged neutropenia, hematological malignancies, stem cell and solid organ transplantation. In critically ill, nonimmunosuppressed patients, severe influenza is recognized as a risk factor for IPA [1]. Recently, severe COVID-19 pneumonia has also been identified as a risk factor [2].

The diagnosis of COVID-19-associated pulmonary aspergillosis (CAPA) is challenging, as COVID-19 patients admitted to the intensive care unit (ICU) commonly lack the host factors that classically predispose them to invasive aspergillosis. A thorough diagnostic work-up, including invasive procedures, is needed in order to discriminate invasive infection from airway colonization. Furthermore, imaging characteristics of IPA are not easily recognized in patients with severe COVID-19 pneumonia, while mycology results need to be interpreted cautiously. Biomarkers, namely serum galactomannan (GM) as well as serum β-D glucan (BDG) have several limitations, as serum GM and BDG, respectively, lack sensitivity and specificity and GM in respiratory samples is only validated for BAL fluid. Different diagnostic criteria and algorithms have been employed in the literature for the definition of CAPA [3]. The EORTC/MSGERC (European Organization for Research and Treatment of Cancer and Mycoses Study Group Education and Research Consortium) criteria [4], the AspICU [5], the modified AspICU algorithm [1] and the IAPA criteria [6], as well as several modifications on these criteria were used in order to classify patients as proven, probable or putative CAPA. In December 2020, a panel of experts endorsed by the European Confederation of Medical Mycology (ECMM) and International Society for Human and Animal Mycology (ISHAM), proposed a case definition for CAPA that is currently adopted by most studies [7]. A newer and simpler taskforce report on the diagnosis and treatment of CAPA became available in June 2021 [8]. Poor outcomes have been related to CAPA, thus early diagnosis and prompt treatment are essential. 

This review aims to present the current status of the literature concerning the incidence, risk factors, outcome, pathophysiology, diagnostic tools/criteria and treatment of CAPA, and ends by comparing influenza-associated pulmonary aspergillosis (IAPA) and CAPA.

## 2. Methods

We performed a qualitative review of the available literature on CAPA. To gather this literature, we used the search string “((COVID) OR (SARS-CoV2) OR (SARS-CoV-2)) AND (Aspergill*)” in Pubmed, with which 273 results were obtained until 12 October 2021. One additional paper in preprint was added through a reference search in these articles, and this paper was published during the writing process of this review [9]. 

Only observational trials, interventional trials specifically for CAPA, or case series (the latter consisting of ≥5 CAPA patients) were considered for inclusion in the tables and graphs in this review. An exception was made for the systematic review by Kula et al. [10], as this incorporated new data based on published papers. 

Papers not mentioning aspergillosis specifically (but only “fungal infections”), not containing clinical information (e.g., the extensive paper of Borman et al. [11]), not specifying which respiratory samples were used for the diagnosis of CAPA (e.g., the paper of Rothe et al. [12], Ichai et al. [13]) or not specifying how many IPA patients were COVID-19 patients (e.g., the paper of Mulet et al. [14]), were excluded as input for the tables and graphs. 

For statistical analyses, differences based on a two-sided hypothesis were assessed using the Chi-square test for categorical data in IBM SPSS Statistics for Windows version 28 (IBM Corp., Armonk, NY, USA).

## 3. Incidence

**Figure 1:**
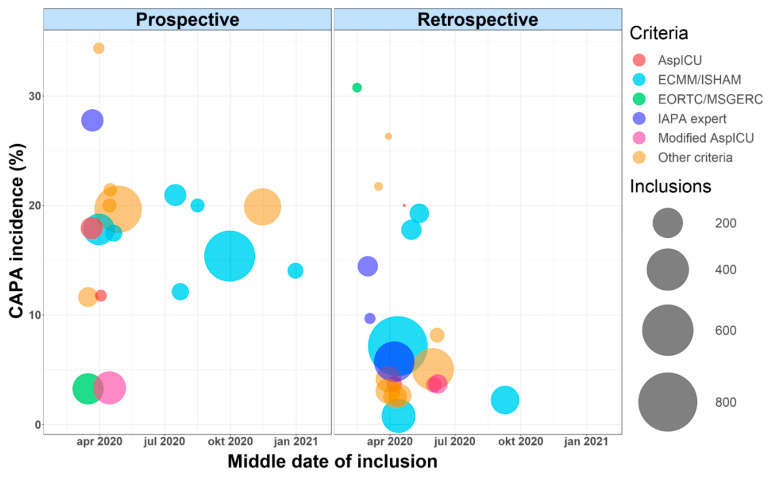
CAPA incidence along a time axis in prospective and retrospective studies with observational data. The time axis is according to the middle date of inclusion (which is the date in the middle, between the first and last day of inclusion in the study). Each dot represents a study. The color of the dot represents the criteria used in the study to diagnose CAPA, while the size of the dot is determined by the number of inclusions in the study. Studies without information on the time period of inclusion as well as case series are excluded from this figure. The partially retrospective/prospective study by Janssen et al. [16] is counted as a retrospective study. CAPA incidence is calculated per study for the combination of proven, probable, putative and possible CAPA cases. The middle date of inclusion is calculated as the date in the middle, between the start and end date of study inclusion.

In total, we summarized data from 7047 patients included in 48 studies (Table S1) [2,9,10,15,16,17,18,19,20,21,22,23,24,25,26,27,28,29,30,31,32,33,34,35,36,37,38,39,40,41,42,43,44,45,46,47,48,49,50,51,52,53,54,55,56,57,58,59]. To review the incidence of CAPA in severe COVID-19, we used data reported by 41 studies with observational data, with a total number of 6193 included patients (Table 1 and Table S1). The reported CAPA incidence is widely variable among purely observational studies, ranging from 0.7 to 34.4% among patients with severe COVID-19 [25,31]. Overall, we found a CAPA incidence (proven, probable or putative) of 10.9% among 6193 COVID-19 patients in observational studies (11.1% among 5904 patients in ICU-only trials) (Table 1 and Table S1). This incidence rises to 12.9% when adding possible CAPA numbers. Putting the reported CAPA incidences of all included studies along a time-axis, by taking the middle date of inclusion per study (i.e., the date between the start and end date of inclusion), we could not observe a clear time–incidence association (Figure 1).

**Figure 2:**
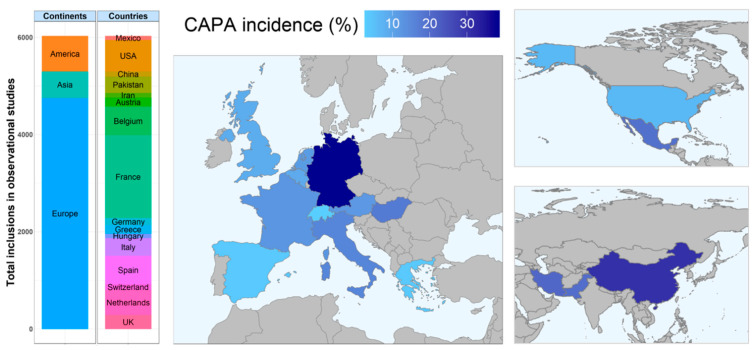
Regional variation in number of inclusions and CAPA incidence in studies with observational data. Bar chart on the left shows the number of patients included in studies with observational data on CAPA, either per continent or per country. The maps on the right show the pooled CAPA incidence per country in studies with observational data. Only studies with exact information on the countries where patients were recruited were included in this figure [2,9,15,16,18,20,21,22,23,24,25,26,27,28,29,30,31,32,33,35,36,37,38,40,41,43,44,45,47,48,49,51,52,53,54,55,56,57,58,59]. CAPA incidence is calculated per study for the combination of proven, probable, putative and possible CAPA cases.

Several factors caused a wide variability in the incidence of CAPA in retrospective (and to a lesser extent prospective) observational trials. The first factor is a regional difference in CAPA incidence, e.g., reported incidences have been as low as 3.3% in Greece and as high as 38.7% in Germany (Figure 2) [2,18,22,31]. Whether this is solely caused by regional differences in test strategies (further discussed below), or also by other regional factors, such as differences in circulating SARS-CoV2 strains, deserves further investigation. Strikingly, these differences do not seem to correspond with differences in the IPA incidence seen in well-known at-risk patient populations (for instance, reported to be higher for Greece than for Germany) [60]. In general, most observational studies investigating CAPA incidence have been performed in European countries. Of the 5953 patients included in observational studies of whom we have data on the country of inclusion, 4757 (79.9%) were included in Europe (Figure 2). For the other continents, we only have some data from a small number of studies from the USA, Mexico, China, Iran and Pakistan. For most of Asia, North- and South-America, Africa and Oceania we have no clue on the CAPA incidence. Cases have been reported in these continents (e.g., the case report from Sharma et al. [61], describing a CAPA case in Australia), but observational studies from these continents would be more than welcome to get a better grasp on the worldwide burden of CAPA. 

A second factor was the heterogeneity of the diagnostic criteria used, especially in the beginning of the pandemic, as it was only in December 2020 that the ECMM/ISHAM CAPA consensus criteria were published [7]. 

A third factor is the wide range of types of respiratory samples used in clinical practice for mycological diagnostics. During the COVID-19 pandemic, there was a surge in the reliance on nonbronchoscopic lavage (NBL) and tracheal aspirates to perform the fungal work-up rather than bronchoalveolar lavage, due to safety concerns [8]. Most of the fungal diagnostic tools have not been validated for these types of respiratory specimen, and using them may, as such, have led to an overestimation of the CAPA incidence (misidentifying colonization and invasion) [62]. 

A fourth factor is the highly variable rate of fungal diagnostic work-up, especially in retrospective trials. A low rate of fungal work-up via lower respiratory tract sampling will inevitably lead to a lower CAPA case finding. For example, the CAPA incidence was only 5% in the retrospective trial of Dellière et al., but this incidence rose to 19% when only including patients who underwent lower respiratory tract sampling for fungal work-up [41]. This leads to the hypothesis that the total incidence might have been higher if all patients had received lower respiratory tract sampling. However, only a few published studies have thoroughly described how many patients received at least one lower respiratory tract sample. Moreover, not only the rate, but also the types of analysis used in the fungal work-up play a role: for instance, in some studies, non-culture-based diagnostic tools such as GM testing are not performed. This variation in the extent of analysis types used in the fungal diagnostic work-up thus serves as a fifth factor that may lead to an underestimation of the CAPA incidence [63]. 

Finally, a sixth factor is the type of study design: retrospective or prospective observational. Strikingly, when looking at prospective observational trials only, the incidence of CAPA (proven, probable or putative) in ICU-admitted patients is 15.1% among 2953 included patients while only being 7.1% among 2951 patients included in retrospective observational trials (Figure 1, Table 1 and Table S1). When adding possible CAPA according to the ECMM/ISHAM-criteria to these numbers, the CAPA incidence in prospective studies rises to 17.4% and to 8.3% in retrospective studies. The raised awareness leading to a more thorough fungal work-up in severe COVID-19 patients in prospective trials may, at least in part, have led to this discrepancy. 

Proven cases of CAPA are generally rare, as the systematic review by Kula et al. reported an incidence of only 1.2% in 677 patients [10]. However, the prospective autopsy study by Fortarezza et al. diagnosed proven CAPA in 9 of 45 (20%) deceased COVID-19 patients [24]. Sampling errors and an antifungal treatment-related disappearance of the aspergillosis by the time a biopsy or autopsy is performed, may potentially have caused these low rates of proven CAPA in the autopsy literature when compared with the rates of probable/putative CAPA. 

CAPA is typically diagnosed approximately one week after ICU admission. The median time between ICU admission and the first positive mycological evidence was 6 days (IQR 3–9 days) in the largest observational study by Janssen et al. [16]. Since most people with severe COVID-19 require ICU admission at approximately 10 days after symptom onset [64,65,66,67], CAPA occurs late during the COVID-19 disease course.

One specific manifestation of CAPA is invasive *Aspergillus* tracheobronchitis (IATB), an entity recognized in IAPA as well. In IATB, plaques, pseudomembranes or ulcers caused by *Aspergillus* are seen bronchoscopically. Reports on CAPA IATB are scarce, probably in part due to a reluctance to perform bronchoscopy in severe COVID-19 patients due to safety concerns (especially in the first wave of the pandemic) [68]. In the paper by Janssen et al., CAPA IATB was reported in 4 of 90 CAPA patients (4.4%) [16]. 

## 4. Risk Factors

Risk factors for CAPA are not well defined. Classic EORTC/MSGERC host factors for IPA in immunocompromised patients, including neutropenia and prolonged use of corticosteroids, are not routinely found in patients with COVID-19 pneumonia who are complicated with *Aspergillus* infection. Only one of the five studies that specifically looked for the presence of EORTC/MSGERC host factors could show a significant association with CAPA [16,17,37,56,59].

Overall, more severely ill ICU-admitted patients with comorbidities, seem to be more susceptible. CAPA is extremely rare in nonsevere COVID-19 cases, thus, only severe COVID-19 can be seen as a risk factor for CAPA. As such, several studies have shown an association between CAPA incidence and severity scores (such as APACHE II and SOFA) on ICU admission [9,23,31,37], while others have shown this for mechanical ventilation [18,37,49]. 

**Figure 3:**
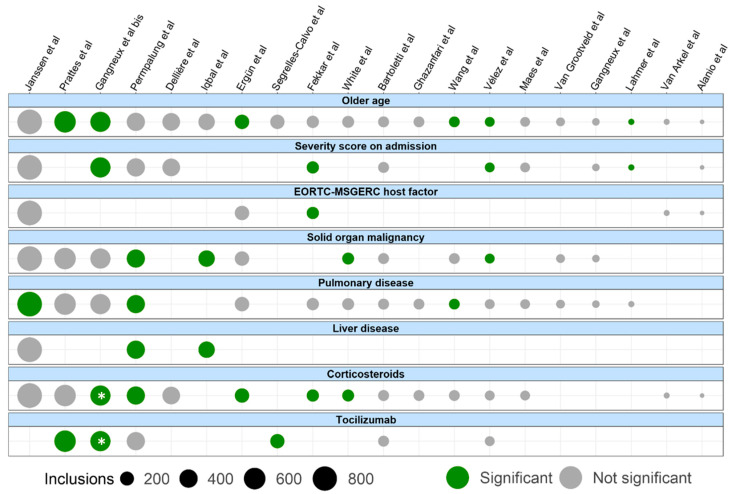
Selected risk factors among the studies in which these data were available. References of cited studies are: [9,16,17,18,21,23,26,31,32,33,37,38,41,44,47,51,54,56,57,59]. Only risk factors that were assessed by multiple studies with observational data are displayed, and only studies with assessments of at least two displayed risk factors are shown. The color indicates whether a significant association was found between CAPA and the risk factor in the study. Size of the dot indicates the number of inclusions in the study. Remarks: * For the study by Gangneux et al. [9], corticosteroids and tocilizumab were only significantly associated with the development of CAPA when combined. For those studies reporting a significant relationship between pulmonary disease and CAPA, this was COPD for Janssen et al. (only in the discovery cohort consisting of 519 patients) [16] and Wang et al. [57], pulmonary vascular diseases for Permpalung et al. [32], and chronic respiratory illness (not specified) for White et al. [47].

Several other patient factors have been implicated, although not unequivocally. Examples are age [16,17,18,23,57], (active) solid organ malignancy [21,32,47] and pulmonary disease [16,32,57], though most studies investigating these factors could not find a significant association with CAPA (Figure 3). Only three studies reported the number of included patients with liver disease (Figure 3), of which two could show an association with the development of CAPA in a univariate analysis [21,32].

Corticosteroids are recognized as a risk factor for invasive fungal infections, including aspergillosis, and are currently recommended for the treatment of severe COVID-19 [7]. Several studies have highlighted an association between different kinds of corticosteroid use (either alone or in combination with anti-IL6 blockade) in severe COVID-19 and *Aspergillus* infection [9,17,32,37,47], though many others (including the two largest prospective studies to date) were unable to corroborate this association (Figure 3). These discrepancies may be related to different cumulative doses of corticosteroids, or due to a lack of statistical power [41], especially in those studies that recruited (part of their) patients after the preprint publication of the RECOVERY trial in June 2020, supporting dexamethasone in severe COVID-19 [69]. In this respect, all prior mentioned studies that showed an association between corticosteroid use and CAPA had a ‘middle date of inclusion’ (the date in the middle between the start and end date of study inclusion) before 31 May 2020. Eventually, a group of 28 international experts provided a taskforce report on the diagnosis and management of CAPA that does not recommend the discontinuation of dexamethasone or corticosteroids for the treatment of severe COVID-19 [8].

Several studies have shown that anti-interleukin-6 receptor treatment increases the risk of CAPA [18,44,45]. However, this association needs further inspection, as not all studies were able to show a significant association between tocilizumab and CAPA incidence [15,23,32,51], while the large study by Gangneux et al. could only show a statistically significant association with CAPA for patients treated with a combination of corticosteroids and tocilizumab [9]. 

Interestingly, Segrelles-Calvo et al. reported a significant association between therapy with type 1β IFN and CAPA [44]. However, no other studies have assessed whether such an association exists.

Only a few studies looked at the risk that (broad-spectrum) antibiotics might confer to CAPA development. The studies of Dellière et al. [41] and Wang et al. [57] found an association with CAPA development for a cumulative azithromycin dose of ≥1500 mg and beta-lactamase inhibitor therapy, respectively. However, Lahmer et al. [31] and White et al. [47] could not find an association between antibiotics and CAPA incidence, while Janssen et al. [16] could not corroborate the association between azithromycin and CAPA. Regarding bacterial co-infections, only Vélez et al. have reported that CAPA patients more often had bacterial isolates found in their central venous cultures, while they could not show this for endotracheal tube cultures or sputum cultures [23]. 

Finally, negative pressure in ICU rooms, implemented during the pandemic in order to decrease the risk of SARS-CoV2 transmission, may favor air contamination with *Aspergillus*. Ichai et al. reported a high incidence of *Aspergillus fumigatus* infection and colonization that was associated with negative pressure in ICU rooms and was countered effectively by switching to neutral or slightly positive pressure and antifungal prophylaxis [13].

To conclude, how much COVID-19 severe pneumonia in itself predisposes patients to invasive aspergillosis and what the contribution is of other risk factors, such as corticosteroid treatment, is still not completely clear and thus deserves further investigation. 

For a subset of risk factors, data per observational study for which these data are available is represented in Figure 3.

## 5. Outcome

CAPA was associated with a higher mortality and/or increased length of stay in 12 studies with a total of 2568 included patients [9,15,17,18,21,31,33,37,41,44,47,53]. All-cause mortality was 55.2% in 728 CAPA patients included in observational studies with mortality data available, excluding autopsy studies (Table 2). This percentage must be interpreted cautiously, as criteria for CAPA diagnosis and mortality endpoints (e.g., ICU discharge, 90 days after ICU admission, etc.) vary among the different studies (Table S2). For studies with clear mortality data of both CAPA patients and patients without arguments for CAPA nor *Aspergillus* colonization, the mortality was significantly higher in the CAPA group (46.2% in 539 CAPA patients) than in the non-CAPA group (31.3% of 3237 patients) (*p* < 0.001) (Table 2). In multivariable logistic regression analyses, the studies by Bartoletti et al. and by Prattes et al. could distinguish CAPA as independently associated with a higher mortality [18,51], a finding that was not corroborated by the study by Janssen et al. [16]. 

We only found five studies that looked into the impact of antifungal treatment on survival in CAPA patients specifically [9,17,36,48,51]. All found a slightly lower mortality in those treated for CAPA, though none could find a significant difference. A lack of power could be the cause of this, as only 220 CAPA patients in total were included in these studies. 

Further investigation is needed to determine the CAPA contribution to mortality and whether antifungal treatment can improve the prognosis of these patients [33].

## 6. Hypotheses on the Pathophysiology

Since *Aspergillus* is omnipresent, each person is continuously exposed to inhaled *Aspergillus* conidia (spores), which are normally efficiently cleared [70]. Historically, invasive aspergillosis was recognized only in patients with a severe immunocompromised status such as severe prolonged neutropenia. Only in the past two decades, have other patient groups without clear EORTC/MSGERC host risk factors, such as cirrhosis patients, been identified to be at risk for aspergillosis [71]. This seems to be especially the case for patients with severe influenza or COVID-19. Why these specific viral infections lead to an increased incidence of aspergillosis is currently not known, as pathophysiological research looking into this question is currently lacking. 

The airway defense system against invasive fungal disease consists of two major components: the epithelium and the immune system. The epithelium fulfills not only a barrier role, but also releases cytokines, chemokines and antimicrobial proteins [72,73]. For both influenza and COVID-19, virus-induced airway epithelial damage with resulting impaired mucociliary clearance may play a role in the higher susceptibility to aspergillosis. However, since both pneumococcal pneumonia and acute respiratory distress syndrome (ARDS) not caused by influenza/COVID-19, do not lead to a similar incidence of aspergillosis (incidences respectively 5% of 315 patients in the paper by Schauwvlieghe et al. [1], and 8.3% in 423 patients in the paper by Contou et al. [74]), the immune response against *Aspergillus* must be affected as well in IAPA and CAPA. 

The immune response against *Aspergillus* relies mainly on macrophages and neutrophils to phagocytize *Aspergillus* conidia, which are then killed via fusion of the phagosome with lysosomes in a process called LC3-associated phagocytosis [75]. If germinating conidia escape this killing process, they may establish hyphae which are mainly targeted by neutrophils via the production of reactive oxygen species (ROS), antimicrobial peptides and neutrophil extracellular trap (NET) formation [76]. Intrinsic immune dysregulation caused by the influenza virus or by SARS-CoV2 may give *Aspergillus* a chance to establish invasive aspergillosis, though the exact mechanism through which this may occur is not yet known. The impaired type I and III interferon (IFN) response seen in severe COVID-19 has been proposed as a contributing factor for the development of CAPA, as type I IFN promotes T-helper type 1 responses against *Aspergillus* and drives type III IFN production, which promotes neutrophils to act against *Aspergillus fumigatus* [73,77,78]. Impaired cellular immunity may be implicated in CAPA pathogenesis as well, as alveolar macrophages (the first immune cells that inhaled conidia encounter) are depleted and T-helper 1 cells (which support antifungal immunity through type II IFN) are severely dysregulated in patients with severe COVID-19 [79]. 

Not only the virus, but also treatment with immunomodulatory agents may play a role in the pathogenesis of CAPA. Corticosteroids, currently standard of care for treating critically ill COVID-19 patients [69], are known to impair LC3-associated phagocytosis and have been identified as a risk factor for the development of IAPA [1,80]. The development of protective T-helper 17 cells and LC3-associated phagocytosis may be inhibited by IL-6 blockade [81,82], and this blockade has come forth as a risk factor for CAPA in several observational trials, either alone [18,45] or when combined with corticosteroids [9,44].

More research is warranted to elucidate the pathogenesis of IAPA and CAPA, as this knowledge is key to finding novel diagnostic/prognostic biomarkers and new key targets for immunomodulatory therapy. Not only the role of the antifungal host response in CAPA, but also possible underlying genetic causes (e.g., similar to several single nucleotide polymorphisms predisposing allogeneic stem cell transplant patients to IPA) and the lower airway microbiota deserve attention.

## 7. Imaging

Severe COVID-19 pneumonia and CAPA share common imaging characteristics. Typical chest CT findings of severe COVID-19 include peripheral bilateral ground glass opacities, with or without consolidation, or the crazy paving pattern. However, according to the Radiological Society of North America classification, atypical presentation can occur with isolated lobar or segmental consolidation, discrete small nodules, lung cavitation and interlobular septal thickening with pleural effusion [83]. 

Radiological characteristics of IPA often described in severely immunocompromised patients, are not always evident in COVID-19 ARDS. Typically, aspergillosis in patients with EORTC/MSGERC host factors is characterized by intrapulmonary nodules surrounded by a halo of ground-glass attenuation (“halo sign”), especially in the angioinvasive form. Cavity formation and the air crescent sign normally appear later in the course of the disease [84]. However, these signs are not sensitive or specific for CAPA [28], in which a consolidation pattern with or without nodules can be the only manifestation [85]. Airway invasive aspergillosis in COVID-19 may occasionally be characterized by tracheal or bronchial wall thickening, while bronchiolitis presents with centrilobular nodules and branching linear or nodular areas of increased attenuation having a “tree-in-bud” appearance [45,84]. 

Among 105 COVID-19 patients with and without invasive mold infections, Ghazanfari et al. found no statistically significant differences in the radiological appearance [26]. In the retrospective study by Reizine et al., all of the 10 CAPA cases presented with diffuse reticular or alveolar opacities, and most had wedge-shaped segmental or lobar consolidation as well. In this study, both bronchial wall thickening and a halo sign were observed only once, while cavitations, air crescent signs, well-circumscribed nodules and tree-in-bud were not seen [28]. Nodules were observed in 4 of 20 CAPA patients described by Marr et al. [39]. In the prospective study by White et al. including 20 CAPA patients, five CAPA patients had cavities, five had nodules, and one had a tree-in-bud pattern on the chest CT [47]. However, certain imaging findings may not be that specific for CAPA, as Li et al. reported that 21.6% (11/51) of COVID-19 patients appeared to have discrete pulmonary nodules, of which nine had a halo sign [86]. The halo sign has also been described in COVID-19 pneumonia without aspergillosis, as it suggests a local infarction related to microthrombosis [7,24]. Whether other cavitating infections (such as sometimes is seen in those caused by *Staphylococcus aureus* or *Pseudomonas aeruginosa*) may have contributed in some cases to these CT findings is often not clear.

Although CT imaging cannot reliably distinguish severe COVID-19 pneumonia from CAPA, it can rule out other possible diagnoses, while the appearance of multiple nodules or cavitation should lead to a further diagnostic work-up for CAPA [8]. Following the ECMM/ISHAM case definition proposed by Koehler et al., only the presence of pulmonary infiltrate or cavitating infiltrate (not attributed to another cause) on a chest CT, is necessary for the diagnosis of possible and probable CAPA [7]. If the CT does not show any infiltrates, noduli or other arguments for aspergillosis, this can be a tool to withhold a CAPA diagnosis in cases where only a respiratory sample other than BAL has a positive mycological argument.

## 8. Mycological Diagnostic Tools

Besides clinical arguments and imaging, mycological evidence is the classical third pillar for the diagnosis of invasive aspergillosis [4]. As the clinical and imaging aspects of aspergillosis in severe COVID-19 patients are often aspecific, the clinician has to rely heavily on mycology to diagnose CAPA [7]. 

### 8.1. Systemic Markers

Systemic markers are well-known and popular, but lack sensitivity for CAPA. As CAPA only becomes angioinvasive in a minority of patients, serum GM (often positive in neutropenic patients) is only rarely positive in CAPA patients: when combining data of CAPA (proven, probable and putative) patients from studies in which serum GM testing was reported, only 80 of 405 (20%) CAPA patients had a positive serum GM (Table S3). Routine screening of severe COVID-19 patients with serum GM is a popular practice, but clinicians must be aware that a negative serum GM does not rule out CAPA. The more sensitive serum BDG lacks specificity for aspergillosis as it is detected in several invasive mold infections, and as such it is not recommended to use for CAPA screening [7]. Serum GM and BDG can however be used as markers for disease severity, as positivity of these tests is associated with an increased mortality in CAPA patients [17]. 

### 8.2. Bronchoscopy

Bronchoscopy has a central position in the work-up of a severe COVID-19 patient suspected to have CAPA. When the pandemic started, clinicians were often reluctant to perform bronchoscopy due to uncertainties regarding the safety of this procedure in times when personal protective equipment was often lacking. However, an increasing body of evidence points towards the minimal risks for both patient and medical personnel if protective measures are taken, and performing this procedure should be standard of care if a patient is suspected of having CAPA or another co-infection [87,88,89,90,91]. 

Visualization of the large airways is the first advantage of performing bronchoscopy. This way, abnormalities associated with IATB, such as white plaques, pseudomembranes or ulcers, can be identified [68]. As finding IATB may impact the choice of therapy for CAPA (e.g., an add-on with aerosolized amphotericin B besides systemic azole therapy), this advantage cannot be underestimated. If such abnormalities are noticed, a biopsy can be performed at once in the absence of contraindications. A biopsy in which septate hyphae invading the tissue are visualized remains the gold standard to prove invasive aspergillosis and is the best way to discriminate colonization from invasive disease [68]. Since most severe COVID-19 patients receive intermediate or high doses of anticoagulants, a biopsy is often not possible. As a safer alternative to biopsy, a brush specimen may be retrieved from a zone with tracheobronchitis [6]. 

### 8.3. Respiratory Samples

Bronchoscopy also allows one to perform bronchoalveolar lavage (BAL) sampling, which provides information on the mycological status of the lower airways and is therefore the superior fluid sample for mycological diagnostics. For BAL-sampling, the instillation of two 20 mL saline volumes is sufficient to retrieve enough fluid for diagnostic testing [63]. The use of NBL has recently been advocated if the means to perform bronchoscopy are lacking (and especially in the beginning of the COVID-19 pandemic, to reduce possible contamination of the bronchoscopist), though only small validation trials for the use of NBL in CAPA diagnosis have been performed and awareness concerning upper airway contamination is required [47,53,92]. The usefulness of NBL in IPA is currently being evaluated (NCT04848831). Data on the validity of tracheal aspirate (TA) or sputum in the diagnosis of IPA are scarce and positive mycological results obtained via these techniques should thus be interpreted with great caution, as they may reflect colonization and not necessarily invasion if there are no other (radiological or mycological) arguments for IPA. When using GM on tracheal aspirates, using a higher cut-off for positivity (2.0) resulted in a higher specificity for CAPA [93]. A prospective study by Van Grootveld et al. conducted in 63 severe COVID-19 patients showed a concordance of 86% between TA and BAL *Aspergillus* PCR and culture [33]. This study suggests that screening via (twice-weekly) TA culture and PCR may be a complementary strategy besides clinical suspicion to identify patients who should receive BAL sampling to look for CAPA. 

Respiratory samples can be used for fungal culture, GM and polymerase chain reaction (PCR) testing. Fungal BAL culture is very specific, but its sensitivity is low (<60%) when looking at autopsy data of critically ill patients with IPA [63]. Besides, a positive culture may be seen in patients with *Aspergillus* airway colonization, and therefore does not always reflect invasion. Solely relying on fungal culture in the work-up for aspergillosis is therefore not sufficient. In the CAPA literature, *A. fumigatus* was the species most commonly isolated (Table S4). 

The detection in BAL fluid of GM, an extracellular glycoprotein antigen released by *Aspergillus* species upon growth, has an excellent sensitivity and specificity in critically ill patients (87% and 88% respectively, for a GM optical index of ≥0.5 in the study by Meersseman et al., including 26 autopsy-proven IPA patients) [63]. Debate concerning the optimal cut-off for the GM optical index is ongoing, but is generally put at 1.0 to increase specificity with a minor reduction of sensitivity [6,94]. As GM testing is not available in all clinical settings, and since batch testing to save costs may cause long delays in the availability of results, mannan-based lateral flow assays (LFA) may serve as a fast and reliable alternative and have been validated in critically ill COVID-19 patients [95,96]. 

PCR testing for *Aspergillus* species in BAL fluid is being increasingly used. A combination of BAL culture, GM and PCR could increase the specificity to diagnose CAPA, though this hypothesis needs validation in large cohorts [54]. 

The importance of performing proper BAL sampling as part of the routine work-up for invasive aspergillosis in the ICU must be emphasized. Studies reporting very low incidences of aspergillosis in ICU patients typically have very low rates of bronchoscopy or BAL sampling, or do not include the use of BAL GM testing to diagnose IPA [97,98,99]. An important issue confounding our knowledge on CAPA incidence is the lack of reporting on the fraction of patients that received a proper fungal work-up (i.e., at least one BAL sampling for fungal culture and GM testing). For example, only 11 out of 41 observational studies report the total number of included patients in whom at least one BAL GM test was performed (Table S3). For future studies, the paper by Yusuf et al. can be taken as a nice example of the proper reporting of this fraction [34]. The message is simple: you can only find aspergillosis in the ICU patient if you are actively looking for it.

## 9. Diagnostic Criteria

The mycological techniques described above translate into various criteria for the diagnosis of CAPA. The widely accepted EORTC/MSGERC criteria, developed with immunocompromised patients in mind, require a specific radiological factor (e.g., halo sign, cavity or air-crescent sign), a host factor (e.g., severe prolonged neutropenia) and a mycological factor (e.g., positive serum GM) for the diagnosis of invasive aspergillosis. These criteria are focused on immunocompromised patients, and are thus generally less useful in critically ill patients who often do not have a pre-existing host factor for the diagnosis of probable IPA [4]. Autopsy studies have shown that adhering too strictly to host factors leads to missed diagnoses of IPA [71,100]. To address this group of critically ill patients more properly, the AspICU algorithm was developed by Blot et al. [5]. This algorithm is more lenient than the EORTC/MSGERC criteria regarding radiological signs, and does not require host factors to be present if a BAL culture is positive and a cytological smear shows branching hyphae. However, an underestimation of the incidence of IPA in the critically ill may arise using this algorithm, as it does not count a positive BAL or serum GM as a sufficient mycological criterion. To tackle this issue, a modified AspICU algorithm was proposed by Schauwvlieghe et al. to diagnose IAPA [1]. This algorithm adds a positive GM in BAL fluid (cut-off 1.0) and serum (cut-off 0.5) to the classical mycological criteria (BAL culture, direct microscopy, histopathology) that suffice for the diagnosis of IAPA in the absence of host factors other than a concurrent severe influenza infection with pulmonary infiltrates. Importantly, in the study by Schauwvlieghe et al., 30 of the 83 patients with IAPA according to the modified AspICU algorithm were not classifiable as putative aspergillosis according to the original AspICU algorithm as they had a positive BAL GM test but a negative lower respiratory tract culture. The modified AspICU algorithm was eventually used as a base for the development of the expert IAPA consensus case definition published in 2020 [6]. 

All the criteria mentioned above (as well as numerous variations on these criteria) have been used in the literature to diagnose CAPA (Figure 1, Table S1). This has contributed to the heterogeneity in reported CAPA incidences. The first major expert CAPA case definition classifying patients as possible, probable or proven CAPA, was published in December 2020 by the European Confederation for Medical Mycology (ECMM) and the International Society for Human and Animal Mycology (ISHAM) [7]. This definition was the first ICU-specific one to include an *Aspergillus* PCR on BAL fluid or serum as mycological tools to diagnose probable CAPA. Moreover, it offers the possibility to diagnose “possible” CAPA via mycological testing on NBL samples. More recently, in June 2021, a taskforce report on the diagnosis and management of CAPA was published by 28 experts in the field [8]. This report clearly states that bronchoscopy with BAL should remain the cornerstone of CAPA diagnosis and removes the possibility of using NBL in its CAPA criteria. It significantly simplifies the diagnostic classification of CAPA, leaving behind the ‘possible’ label introduced by the ECMM/ISHAM definition. Positive BAL microscopy, BAL GM, BAL *Aspergillus* PCR, BAL culture or serum GM suffice for the diagnosis of CAPA in severe COVID-19 patients who are clinically deteriorating without another clear explanation. This CAPA definition, as stated by the taskforce report (or alternatively the ECMM/ISHAM criteria), should be advocated for use in future trials to incorporate homogeneity in the literature. 

## 10. Treatment

Voriconazole or isavuconazole are the first-line antifungals recommended by the ECMM/ISHAM for CAPA [7]. The majority of patients reported in the literature were treated with voriconazole, while in three studies isavuconazole was preferred (Table S3) [13,22,45]. 

Voriconazole and isavuconazole have mainly been evaluated in immunocompromised patients. Voriconazole is metabolized by cytochrome P450 enzymes CYP2C19, CYP2C9, and CYP3A4, resulting in multiple drug–drug interactions [101]. The plasma concentrations should be closely monitored as exposure in critically ill patients is highly variable, especially in those treated with ECMO [102]. Both subtherapeutic and toxic levels have been detected in critically ill patients, resulting in a higher probability of therapeutic failure and neuro- and hepatotoxicity, respectively [8]. A plasma trough concentration of 2–6 mg/L is recommended. However, a delay was observed in reaching voriconazole therapeutic levels (2–6 mg/L) in CAPA patients, as Reizine et al. reported that the therapeutic range was achieved at day 7, with 83.3% of patients having subtherapeutic levels (<2 mg/L) at day 5 [28]. Dexamethasone, which is now the mainstay of treatment in severe COVID-19, is implicated by inducing several CYP450 enzymes and decreasing voriconazole levels [101,103]. Another explanation is that dexamethasone reduces inflammation. Inflammation (as reflected by CRP or IL-6 levels) has been shown to lead to phenoconversion leading to a reduction in CYP450-mediated metabolism. In reverse, glucocorticoids are thought to cancel this effect, leading to a reduced exposure [104,105,106].

Difficulties encountered with the dose adjustment of voriconazole, render isavuconazole a more attractive option [28]. Isavuconazole is considered safer as it does not seem to have a narrow therapeutic window, has less toxicity and fewer drug–drug interactions [107,108]. However, its variability in exposure and the potential impact on efficacy and toxicity in ICU patients has to be determined [8]. Liposomal amphotericin B is the first-line alternative treatment propagated by the ECMM/ISHAM [7]. Nephrotoxicity is the main consideration when liposomal amphotericin B is used as an alternative treatment, especially in COVID-19-related acute kidney injury [7]. Posaconazole or echinocandins are alternative second-line regimens. Posaconazole is currently not propagated as a first-line therapy in the ECMM/ISHAM guideline, despite the head-to-head non-inferiority compared to voriconazole [7,109]. Echinocandins (micafungin or caspofungin) should be considered as salvage agents [7,110]. Triazoles or intravenous lipid formulations of amphotericin B are also indicated for the invasive forms of tracheobronchial aspergillosis, while voriconazole is also the first choice for *Aspergillus flavus*, which is the second most common etiological agent [110]. The duration of treatment should be based on the clinical, radiological and mycological response, but is typically 6–12 weeks [7,110]. 

Environmental triazole resistance (TR_34_/L98H mutation) has been detected in *Aspergillus* strains of CAPA patients [11,36]. These patients were treated with a combination of echinocandin and voriconazole or liposomal amphotericin B. In regions with a high prevalence of azole resistant strains, empiric treatment combining voriconazole or isavuconazole with an echinocandin or liposomal amphotericin B should be administered initially until the susceptibility is known [7,36]. 

New antifungals, such as opelconazole, fosmanogepix, ibrexafungerp, olorofim and rezafungin are currently being evaluated and may be added to the therapeutic armamentarium in the future [7,111].

To date, two studies investigating antifungal prophylaxis for CAPA have been published [15,19]. Hatzl et al. administered mainly posaconazole in 132 ICU COVID-19 patients and found a decreased incidence of CAPA, but without a difference in mortality [15]. Posaconazole is recommended for antifungal prophylaxis in patients with hematological malignancies with a high risk of infection. It exhibits fewer drug–drug interactions compared with voriconazole as it is metabolized predominantly through the uridine diphosphate-glucuronyltransferase enzyme pathways. Other agents used for prophylaxis include voriconazole, itraconazole or inhaled amphotericin B [110]. However, use of itraconazole is less propagated due to its erratic absorption [112,113]. In a retrospective observational study by Van Ackerbroeck et al., prophylaxis with inhaled liposomal amphotericin B twice weekly significantly reduced the incidence of CAPA and IATB in mechanically ventilated COVID-19 patients. The inhalation of liposomal amphotericin B was not associated with adverse events such as bronchospasm [19]. Prophylaxis with isavuconazole to prevent CAPA is currently being studied (NCT04707703). Whether antifungal prophylaxis may have a role in certain COVID-19 populations in order to prevent CAPA and reduce mortality must be further investigated. 

## 11. Comparison of IAPA and CAPA

**Figure 4:**
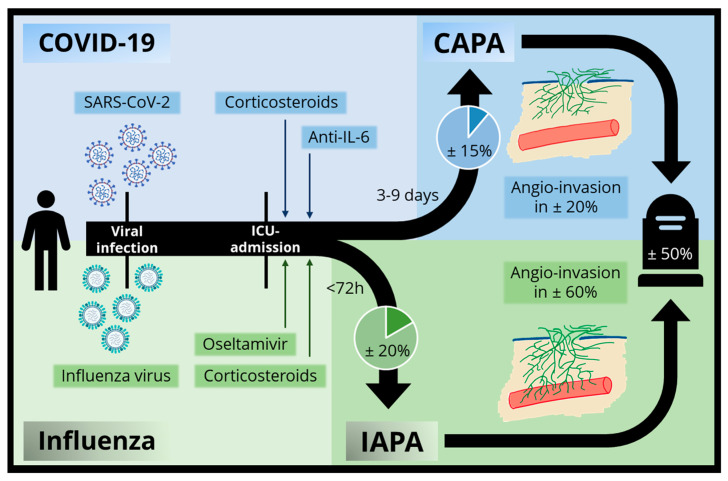
Comparison between IAPA and CAPA. Infograph on the similarities and differences between CAPA and IAPA. Both co-infections occur in ICU-admitted patients. Corticosteroids and anti-IL-6 therapy have been implicated in CAPA pathophysiology, while this is the case for corticosteroids and oseltamivir in IAPA. IAPA tends to occur earlier, more frequently and with more frequent angioinvasion than CAPA. Both lead to an increased mortality. Created with the aid of BioRender.com.

The experience gained with IAPA during the past decade is one of the reasons why CAPA was soon recognized as an important entity in severe COVID-19. Despite being the newer entity, more and larger (prospective) trials have been performed on CAPA than on IAPA, as prospective IAPA research was hampered by the (near complete) worldwide disappearance of severe influenza since the start of the COVID-19 pandemic [114]. Several similarities exist between both entities: they both occur in critically ill patients with severe respiratory distress, are associated with an increased mortality, and as in CAPA, a wide (regional and probably at least partially test strategy-related) variation in IAPA incidence has been described [97,115,116]. However, there are some major differences (Figure 4). First of all, the incidence of IAPA seems to be slightly higher, averaging approximately 20% in most observational studies and reaching even more than 30% in patients with EORTC/MSGERC host-factors [1,117,118,119]. In contrast to CAPA, IAPA is an early phenomenon, with >70% of the cases already being present within the first 48 h of ICU admission [119]. Moreover, IAPA tends to be more aggressive, as indicated by the higher incidence of positive serum GM (generally in >50% of tested IAPA patients) [1,117]. Moreover, most studies point out a clear link between the use of corticosteroids and IAPA-incidence [1,118], while this association is less outspoken in CAPA (Figure 3). 

## 12. Conclusions

CAPA is a severe co-infection affecting critically ill COVID-19 patients, leading to worse outcomes in these patients. The incidence generally ranges between 10% and 15%, though several factors, such as variation in the prospective/retrospective study design, the number of included patients that received an adequate fungal work-up, and the extent of mycological tools used may have caused significant variability in the observed incidences. The treatment of COVID-19 with corticosteroids and/or tocilizumab potentially confer the biggest risk for developing CAPA. Since imaging findings are generally aspecific, proper culture- and non-culture-based mycological testing (preferably using bronchoscopy and bronchoalveolar lavage) is the cornerstone of diagnosis, with the ECMM/ISHAM CAPA case definition or the simpler taskforce CAPA criteria being the advocated tools to classify patients. Azoles are the first-choice antifungal drugs for the treatment of CAPA. 

Despite the availability of plenty of retrospective and prospective observational trials on CAPA, there is still a lot to learn. Incidence data from continents other than Europe are needed to get a better picture of the global burden of this disease. New studies must keep in mind proper reporting of the exact fungal work-up that included patients received. We urge the scientific and medical community to dive deeper into the pathophysiology, diagnosis and therapeutic approach for CAPA, as it has probably contributed substantially to the millions of deaths the COVID-19 pandemic has caused. A better awareness and understanding of this disease will not only prove to be useful in the current pandemic, but will undoubtedly contribute to our knowledge of IAPA as well, and give us a head-start concerning mycological co-infections in the respiratory virus pandemics to come. 

## Figures and Tables

**Table 1:** CAPA incidence in observational studies.

	Included Patients (*n*)	Total Proven CAPA (*n*)	Total Probable or Putative CAPA (*n*)	Total Possible CAPA (*n*)	Total Number of Patients with Coloniz-ation (*n*)	Percentage Proven, Probable or Putative CAPA among All Included Patients
Observational ^1^ studies (*n* = 42)	6193	35	638	102	70	10.9%
ICU-only observational ^1^ studies (*n* = 39)	5904	26	631	102	70	11.1%
ICU-only retrospective (or partially prospective) observational ^1^ studies (*n* = 21)	2951	12	198	35	33	7.1%
ICU-only prospective observational studies (*n* = 18)	2953	14	433	67	37	15.1%

^1^ Case series that mentioned observational data were counted as observational studies. CAPA = COVID-19-associated pulmonary aspergillosis; ICU = intensive care unit. See Table S1 for the full table with data on individual studies.

**Table 2:** Mortality numbers among CAPA and non-CAPA patients in observational trials in which these data were available.

	CAPA Patients ^1^ (*n*)	Number of CAPA Patients ^1^ That Died, All-Cause Mortality (*n*, Percentage)	Total Number of Patients without Arguments for CAPA ^2^	Total Number of Patients without Arguments for CAPA That Died ^3^ (*n*, Percentage)
Observational trials ^2^ (*n* = 37)	728	402 (55.2%)	4522	NA
Observational trials with data on mortality in patients without arguments for CAPA ^3^ (*n* = 21)	539	249 (46.2%)	3238	1014 (31.3%)

Remarks: ^1^ Proven, probable, putative or possible CAPA. ^2^ Only non-autopsy studies with observational data are included in this table; case series that mentioned observational data were counted as observational studies. ^3^ Number of studies that provided clear data on the mortality in patients that did not have arguments for aspergillosis (including no patients explicitly categorized as colonized with *Aspergillus*). For a full version of this table, see Supplementary Table S2.

## Supplementary Materials

The following are available online at https://www.mdpi.com/article/10.3390/jof7121067/s1, Table S1: General information on observational studies and case series with 5 or more CAPA cases publicly available on 12 October 2021, Table S2: Mortality numbers among CAPA and non-CAPA patients in non-autopsy observational trials in which these data were available. Table S3: Data on *Aspergillus* culture and galactomannan. Table S4: Cultured *Aspergillus* species and antifungal treatment.

## References

[1] Schauwvlieghe A.F.A.D., Rijnders B.J.A., Philips N., Verwijs R., Vanderbeke L., Van Tienen C., Lagrou K., Verweij P.E., Van de Veerdonk F.L., Gommers D. (2018). Invasive Aspergillosis in Patients Admitted to the Intensive Care Unit with Severe Influenza: A Retrospective Cohort Study. Lancet Respir. Med..

[2] Koehler P., Cornely O.A., Böttiger B.W., Dusse F., Eichenauer D.A., Fuchs F., Hallek M., Jung N., Klein F., Persigehl T. (2020). COVID-19 associated pulmonary aspergillosis. Mycoses.

[3] Dimopoulos G., Almyroudi M.-P., Myrianthefs P., Rello J. (2021). COVID-19-Associated Pulmonary Aspergillosis (CAPA). J. Intensive Med..

[4] Donnelly J.P., Chen S.C., Kauffman C.A., Steinbach W.J., Baddley J.W., Verweij P.E., Clancy C.J., Wingard J.R., Lockhart S.R., Groll A.H. (2020). Revision and Update of the Consensus Definitions of Invasive Fungal Disease from the European Organization for Research and Treatment of Cancer and the Mycoses Study Group Education and Research Consortium. Clin. Infect. Dis..

[5] Blot S.I., Taccone F.S., Van Den Abeele A.M., Bulpa P., Meersseman W., Brusselaers N., Dimopoulos G., Paiva J.A., Misset B., Rello J. (2012). A Clinical Algorithm to Diagnose Invasive Pulmonary Aspergillosis in Critically Ill Patients. Am. J. Respir. Crit. Care Med..

[6] Verweij P., Rijnders B., Brüggemann R., Azoulay E., Bassetti M., Blot S., Calandra T., Clancy C., Cornely O., Chiller T. (2020). International Expert Review of Influenza-Associated Pulmonary Aspergillosis in ICU Patients and Recommendations for a Case Definition. Intensive Care Med..

[7] Koehler P., Bassetti M., Chakrabarti A., Chen S.C.A., Colombo A.L., Hoenigl M., Klimko N., Lass-Flörl C., Oladele R.O., Vinh D.C. (2021). Defining and managing COVID-19-associated pulmonary aspergillosis: The 2020 ECMM/ISHAM consensus criteria for research and clinical guidance. Lancet Infect. Dis..

[8] Verweij P.E., Brüggemann R.J.M., Azoulay E., Bassetti M., Blot S., Buil J.B., Calandra T., Chiller T., Clancy C.J., Cornely O.A. (2021). Taskforce report on the diagnosis and clinical management of COVID-19 associated pulmonary aspergillosis. Intensive Care Med..

[9] Gangneux J.-P., Dannaoui E., Fekkar A., Luyt C.-E., Botterel F., De Prost N., Tadié J.-M., Reizine F., Houzé S., Timsit J.-F. (2021). Fungal infections in mechanically ventilated patients with COVID-19 during the first wave: The French multicentre MYCOVID study. Lancet Respir. Med..

[10] Kula B.E., Clancy C.J., Hong Nguyen M., Schwartz I.S. (2021). Invasive mould disease in fatal COVID-19: A systematic review of autopsies. Lancet Microbe.

[11] Borman A.M., Palmer M.D., Fraser M., Patterson Z., Mann C., Oliver D., Linton C.J., Gough M., Brown P., Dzietczyk A. (2021). COVID-19-associated invasive aspergillosis: Data from the UK national mycology reference laboratory. J. Clin. Microbiol..

[12] Rothe K., Feihl S., Schneider J., Wallnöfer F., Wurst M., Lukas M., Treiber M., Lahmer T., Heim M., Dommasch M. (2021). Rates of bacterial co-infections and antimicrobial use in COVID-19 patients: A retrospective cohort study in light of antibiotic stewardship. Eur. J. Clin. Microbiol. Infect. Dis..

[13] Ichai P., Saliba F., Baune P., Daoud A., Coilly A., Samuel D. (2020). Impact of negative air pressure in ICU rooms on the risk of pulmonary aspergillosis in COVID-19 patients. Crit. Care.

[14] Mulet Bayona J.V., Tormo Palop N., Salvador García C., Fuster Escrivá B., Chanzá Aviñó M., Ortega García P., Gimeno Cardona C. (2021). Impact of the SARS-CoV-2 Pandemic in Candidaemia, Invasive Aspergillosis and Antifungal Consumption in a Tertiary Hospital. J. Fungi.

[15] Hatzl S., Reisinger A.C., Posch F., Prattes J., Stradner M., Pilz S., Eller P., Schoerghuber M., Toller W., Gorkiewicz G. (2021). Antifungal prophylaxis for prevention of COVID-19-associated pulmonary aspergillosis in critically ill patients: An observational study. Crit. Care.

[16] Janssen N.A.F., Nyga R., Vanderbeke L., Jacobs C., Ergün M., Buil J.B., van Dijk K., Altenburg J., Bouman C.S.C., van der Spoel H.I. (2021). Multinational Observational Cohort Study of COVID-19–Associated Pulmonary Aspergillosis1. Emerg. Infect. Dis..

[17] Ergün M., Brüggemann R.J.M., Alanio A., Dellière S., van Arkel A., Bentvelsen R.G., Rijpstra T., van der Sar-van der Brugge S., Lagrou K., Janssen N.A.F. (2021). Aspergillus Test Profiles and Mortality in Critically Ill COVID-19 Patients. J. Clin. Microbiol..

[18] Prattes J., Wauters J., Giacobbe D.R., Salmanton-García J., Maertens J., Bourgeois M., Reynders M., Rutsaert L., Van Regenmortel N., Lormans P. (2021). Risk factors and outcome of pulmonary aspergillosis in critically ill coronavirus disease 2019 patients—a multinational observational study by the European Confederation of Medical Mycology. Clin. Microbiol. Infect..

[19] Van Ackerbroeck S., Rutsaert L., Roelant E., Dillen K., Wauters J., Van Regenmortel N. (2021). Inhaled liposomal amphotericin-B as a prophylactic treatment for COVID-19-associated pulmonary aspergillosis/aspergillus tracheobronchitis. Crit. Care.

[20] Szabo B.G., Lakatos B., Bobek I., Szabo E., Szlavik J., Vályi-Nagy I. (2021). Invasive fungal infections among critically ill adult COVID-19 patients: First experiences from the national centre in Hungary. J. Med. Mycol..

[21] Iqbal A., Ramzan M., Akhtar A., Ahtesham A., Aslam S., Khalid J. (2021). COVID-Associated Pulmonary Aspergillosis and Its Related Outcomes: A Single-Center Prospective Observational Study. Cureus.

[22] Paramythiotou E., Dimopoulos G., Koliakos N., Siopi M., Vourli S., Pournaras S., Meletiadis J. (2021). Epidemiology and Incidence of COVID-19-Associated Pulmonary Aspergillosis (CAPA) in a Greek Tertiary Care Academic Reference Hospital. Infect. Dis..

[23] Vélez Pintado M., Camiro-Zúñiga A., Aguilar Soto M., Cuenca D., Mercado M., Crabtree-Ramirez B. (2021). COVID-19-associated invasive pulmonary aspergillosis in a tertiary care center in Mexico City. Med. Mycol..

[24] Fortarezza F., Boscolo A., Pezzuto F., Lunardi F., Acosta M.J., Giraudo C., Del Vecchio C., Sella N., Tiberio I., Godi I. (2021). Proven COVID-19—associated pulmonary aspergillosis in patients with severe respiratory failure. Mycoses.

[25] Wasylyshyn A.I., Wasylyshyn G.R., Linder K.A., Miceli M.H. (2021). COVID-19-Associated Pulmonary Aspergillosis at an Academic Medical Center in the Midwestern United States. Mycopathologia.

[26] Ghazanfari M., Arastehfar A., Davoodi L., Yazdani Charati J., Moazeni M., Abastabar M., Haghani I., Mirzakhani R., Mayahi S., Fang W. (2021). Pervasive but Neglected: A Perspective on COVID-19-Associated Pulmonary Mold Infections among Mechanically Ventilated COVID-19 Patients. Front. Med..

[27] Oliva A., Ceccarelli G., Borrazzo C., Ridolfi M., D’Ettorre G., Alessandri F., Ruberto F., Pugliese F., Raponi G.M., Russo A. (2021). Comparison of clinical features and outcomes in COVID-19 and influenza pneumonia patients requiring intensive care unit admission. Infection.

[28] Reizine F., Pinceaux K., Lederlin M., Autier B., Guegan H., Gacouin A., Luque-Paz D., Boglione-Kerrien C., Bacle A., Le Daré B. (2021). Influenza- and COVID-19-Associated Pulmonary Aspergillosis: Are the Pictures Different?. J. Fungi.

[29] Sánchez Martín C., Madrid Martínez E., González Pellicer R., Armero Ibáñez R., Martínez González E., Llau Pitarch J.V. (2021). Aspergilosis pulmonar invasiva en pacientes con síndrome de distrés respiratorio por COVID-19. Rev. Esp. Anestesiol. Reanim..

[30] Ripa M., Galli L., Poli A., Oltolini C., Spagnuolo V., Mastrangelo A., Muccini C., Monti G., De Luca G., Landoni G. (2021). Secondary infections in patients hospitalized with COVID-19: Incidence and predictive factors. Clin. Microbiol. Infect..

[31] Lahmer T., Kriescher S., Herner A., Rothe K., Spinner C.D., Schneider J., Mayer U., Neuenhahn M., Hoffmann D., Geisler F. (2021). Invasive pulmonary aspergillosis in critically ill patients with severe COVID-19 pneumonia: Results from the prospective AspCOVID-19 study. PLoS ONE.

[32] Permpalung N., Chiang T.P.-Y., Massie A.B., Zhang S.X., Avery R.K., Nematollahi S., Ostrander D., Segev D.L., Marr K.A. (2021). Coronavirus Disease 2019–Associated Pulmonary Aspergillosis in Mechanically Ventilated Patients. Clin. Infect. Dis..

[33] Van Grootveld R., van Paassen J., de Boer M.G.J., Claas E.C.J., Kuijper E.J., van der Beek M.T. (2021). Systematic screening for COVID-19 associated invasive aspergillosis in ICU patients by culture and PCR on tracheal aspirate. Mycoses.

[34] Yusuf E., Vonk A., van den Akker J.P.C., Bode L., Sips G.J., Rijnders B.J.A., de Steenwinkel J., Verkaik N.J., Vogel M., van der Eerden M. (2021). Frequency of positive aspergillus tests in COVID-19 patients in comparison to other patients with pulmonary infections admitted to the intensive care unit. J. Clin. Microbiol..

[35] Versyck M., Zarrougui W., Lambiotte F., Elbeki N., Saint-Leger P. (2021). Invasive pulmonary aspergillosis in COVID-19 critically ill patients: Results of a French monocentric cohort. J. Mycol. Med..

[36] Meijer E.F.J., Dofferhoff A.S.M., Hoiting O., Meis J.F. (2021). COVID-19–associated pulmonary aspergillosis: A prospective single-center dual case series. Mycoses.

[37] Fekkar A., Lampros A., Mayaux J., Poignon C., Demeret S., Constantin J.M., Marcelin A.G., Monsel A., Luyt C.E., Blaize M. (2021). Occurrence of invasive pulmonary fungal infections in patients with severe COVID-19 admitted to the ICU. Am. J. Respir. Crit. Care Med..

[38] Maes M., Higginson E., Pereira-Dias J., Curran M.D., Parmar S., Khokhar F., Cuchet-Lourenço D., Lux J., Sharma-Hajela S., Ravenhill B. (2021). Ventilator-associated pneumonia in critically ill patients with COVID-19. Crit. Care.

[39] Marr K.A., Platt A., Tornheim J.A., Zhang S.X., Datta K., Cardozo C., Garcia-Vidal C. (2021). Aspergillosis complicating severe coronavirus disease. Emerg. Infect. Dis..

[40] Razazi K., Arrestier R., Haudebourg A.F., Benelli B., Carteaux G., Decousser J., Fourati S., Woerther P.L., Schlemmer F., Charles-Nelson A. (2020). Risks of ventilator-associated pneumonia and invasive pulmonary aspergillosis in patients with viral acute respiratory distress syndrome related or not to Coronavirus 19 disease. Crit. Care.

[41] Dellière S., Dudoignon E., Fodil S., Voicu S., Collet M., Oillic P.A., Salmona M., Dépret F., Ghelfenstein-Ferreira T., Plaud B. (2021). Risk factors associated with COVID-19-associated pulmonary aspergillosis in ICU patients: A French multicentric retrospective cohort. Clin. Microbiol. Infect..

[42] Benedetti M.F., Alava K.H., Sagardia J., Cadena R.C., Laplume D., Capece P., Posse G., Nusblat A.D., Cuestas M.L. (2021). COVID-19 associated pulmonary aspergillosis in ICU patients: Report of five cases from Argentina. Med. Mycol. Case Rep..

[43] Nebreda-Mayoral T., Miguel-Gómez M.A., March-Rosselló G.A., Puente-Fuertes L., Cantón-Benito E., Martínez-García A.M., Muñoz-Martín A.B., Orduña-Domingo A. (2020). Infección bacteriana/fúngica en pacientes con COVID-19 ingresados en un hospital de tercer nivel de Castilla y León, España. Enfermedades Infecciosas y Microbiología Clínica.

[44] Segrelles-Calvo G., Araújo G.R.S., Llopis-Pastor E., Carrillo J., Hernández-Hernández M., Rey L., Rodríguez Melean N., Escribano I., Antón E., Zamarro C. (2021). Prevalence of opportunistic invasive aspergillosis in COVID-19 patients with severe pneumonia. Mycoses.

[45] Machado M., Valerio M., Álvarez-Uría A., Olmedo M., Veintimilla C., Padilla B., De la Villa S., Guinea J., Escribano P., Ruiz-Serrano M.J. (2021). Invasive pulmonary aspergillosis in the COVID-19 era: An expected new entity. Mycoses.

[46] Mitaka H., Perlman D.C., Javaid W., Salomon N. (2020). Putative invasive pulmonary aspergillosis in critically ill patients with COVID-19: An observational study from New York City. Mycoses.

[47] White P.L., Dhillon R., Cordey A., Hughes H., Faggian F., Soni S., Pandey M., Whitaker H., May A., Morgan M. (2021). A National Strategy to Diagnose Coronavirus Disease 2019–Associated Invasive Fungal Disease in the Intensive Care Unit. Clin. Infect. Dis..

[48] Dupont D., Menotti J., Turc J., Miossec C., Wallet F., Richard J.C., Argaud L., Paulus S., Wallon M., Ader F. (2021). Pulmonary aspergillosis in critically ill patients with Coronavirus Disease 2019 (COVID-19). Med. Mycol..

[49] Sarrazyn C., Dhaese S., Demey B., Vandecasteele S., Reynders M., Van Praet J.T. (2021). Incidence, risk factors, timing, and outcome of influenza versus COVID-19–associated putative invasive aspergillosis. Infect. Control Hosp. Epidemiol..

[50] Falces-Romero I., Ruiz-Bastián M., Díaz-Pollán B., Maseda E., García-Rodríguez J., Montero-Vega M.D., Romero-Gómez M.P., García-Bujalance S., Cendejas-Bueno E., Toro-Rueda C. (2020). Isolation of Aspergillus spp. in respiratory samples of patients with COVID-19 in a Spanish Tertiary Care Hospital. Mycoses.

[51] Bartoletti M., Pascale R., Cricca M., Rinaldi M., Maccaro A., Bussini L., Fornaro G., Tonetti T., Pizzilli G., Francalanci E. (2020). Epidemiology of Invasive Pulmonary Aspergillosis Among Intubated Patients with COVID-19: A Prospective Study. Clin. Infect. Dis..

[52] Nasir N., Farooqi J., Mahmood S.F., Jabeen K. (2020). COVID-19-associated pulmonary aspergillosis (CAPA) in patients admitted with severe COVID-19 pneumonia: An observational study from Pakistan. Mycoses.

[53] Van Biesen S., Kwa D., Bosman R.J., Juffermans N.P. (2020). Detection of Invasive Pulmonary Aspergillosis in COVID-19 with Nondirected BAL. Am. J. Respir. Crit. Care Med..

[54] Gangneux J.P., Reizine F., Guegan H., Pinceaux K., Le Balch P., Prat E., Pelletier R., Belaz S., Le Souhaitier M., Le Tulzo Y. (2020). Is the covid-19 pandemic a good time to include aspergillus molecular detection to categorize aspergillosis in icu patients? A monocentric experience. J. Fungi.

[55] Lamoth F., Glampedakis E., Boillat-Blanco N., Oddo M., Pagani J.-L. (2020). Incidence of invasive pulmonary aspergillosis among critically ill COVID-19 patients. Clin. Microbiol. Infect..

[56] van Arkel A.L.E., Rijpstra T.A., Belderbos H.N.A., van Wijngaarden P., Verweij P.E., Bentvelsen R.G. (2020). COVID-19–associated Pulmonary Aspergillosis. Am. J. Respir. Crit. Care Med..

[57] Wang J., Yang Q., Zhang P., Sheng J., Zhou J., Qu T. (2020). Clinical characteristics of invasive pulmonary aspergillosis in patients with COVID-19 in Zhejiang, China: A retrospective case series. Crit. Care.

[58] Rutsaert L., Steinfort N., Van Hunsel T., Bomans P., Naesens R., Mertes H., Dits H., Van Regenmortel N. (2020). COVID-19-associated invasive pulmonary aspergillosis. Ann. Intensive Care.

[59] Alanio A., Dellière S., Fodil S., Bretagne S., Mégarbane B. (2020). Prevalence of putative invasive pulmonary aspergillosis in critically ill patients with COVID-19. Lancet Respir. Med..

[60] Bongomin F., Gago S., Oladele R., Denning D. (2017). Global and Multi-National Prevalence of Fungal Diseases—Estimate Precision. J. Fungi.

[61] Sharma A., Hofmeyr A., Bansal A., Thakkar D., Lam L., Harrington Z., Bhonagiri D. (2021). COVID-19 associated pulmonary aspergillosis (CAPA): An Australian case report. Med. Mycol. Case Rep..

[62] Prattes J., Koehler P., Hoenigl M., Wauters J., Giacobbe D.R., Lagrou K., Salmanton-García J., Rautemaa-Richardson R., Hatzl S., Maertens J. (2021). COVID-19 associated pulmonary aspergillosis: Regional variation in incidence and diagnostic challenges. Intensive Care Med..

[63] Meersseman W., Lagrou K., Maertens J., Wilmer A., Hermans G., Vanderschueren S., Spriet I., Verbeken E., Van Wijngaerden E. (2008). Galactomannan in Bronchoalveolar Lavage Fluid: A Tool for Diagnosing Aspergillosis in Intensive Care Unit Patients. Am. J. Respir. Crit. Care Med..

[64] Yang X., Yu Y., Xu J., Shu H., Xia J., Liu H., Wu Y., Zhang L., Yu Z., Fang M. (2020). Clinical course and outcomes of critically ill patients with SARS-CoV-2 pneumonia in Wuhan, China: A single-centered, retrospective, observational study. Lancet Respir. Med..

[65] Zhou F., Yu T., Du R., Fan G., Liu Y., Liu Z., Xiang J., Wang Y., Song B., Gu X. (2020). Clinical course and risk factors for mortality of adult inpatients with COVID-19 in Wuhan, China: A retrospective cohort study. Lancet.

[66] Huang C., Wang Y., Li X., Ren L., Zhao J., Hu Y., Zhang L., Fan G., Xu J., Gu X. (2020). Clinical features of patients infected with 2019 novel coronavirus in Wuhan, China. Lancet.

[67] Wang D., Hu B., Hu C., Zhu F., Liu X., Zhang J., Wang B., Xiang H., Cheng Z., Xiong Y. (2020). Clinical Characteristics of 138 Hospitalized Patients With 2019 Novel Coronavirus–Infected Pneumonia in Wuhan, China. JAMA.

[68] Van de Veerdonk F.L., Brüggemann R.J.M., Vos S., de Hertogh G., Wauters J., Reijers M.H.E., Netea M.G., Schouten J.A., Verweij P.E. (2021). COVID-19-associated Aspergillus tracheobronchitis: The interplay between viral tropism, host defence, and fungal invasion. Lancet Respir. Med..

[69] (2021). Recovery Collaborative Group Dexamethasone in Hospitalized Patients with COVID-19. N. Engl. J. Med..

[70] Latgé J.-P., Chamilos G. (2019). Aspergillus fumigatus and Aspergillosis in 2019. Clin. Microbiol. Rev..

[71] Meersseman W., Lagrou K., Maertens J., Wijngaerden E.V. (2007). Invasive Aspergillosis in the Intensive Care Unit. Clin. Infect. Dis..

[72] Bertuzzi M., Hayes G.E., Icheoku U.J., van Rhijn N., Denning D.W., Osherov N., Bignell E.M. (2018). Anti-aspergillus activities of the respiratory epithelium in health and disease. J. Fungi.

[73] Dewi I.M., Janssen N.A., Rosati D., Bruno M., Netea M.G., Brüggemann R.J., Verweij P.E., van de Veerdonk F.L. (2021). Invasive pulmonary aspergillosis associated with viral pneumonitis. Curr. Opin. Microbiol..

[74] Contou D., Dorison M., Rosman J., Schlemmer F., Gibelin A., Foulet F., Botterel F., Carteaux G., Razazi K., Brun-Buisson C. (2016). Aspergillus-positive lower respiratory tract samples in patients with the acute respiratory distress syndrome: A 10-year retrospective study. Ann. Intensive Care.

[75] Sprenkeler E.G.G., Gresnigt M.S., van de Veerdonk F.L. (2016). LC3-associated phagocytosis: A crucial mechanism for antifungal host defence against Aspergillus fumigatus. Cell. Microbiol..

[76] Van De Veerdonk F.L., Gresnigt M.S., Romani L., Netea M.G., Latgé J.P. (2017). Aspergillus fumigatus morphology and dynamic host interactions. Nat. Rev. Microbiol..

[77] Gafa V., Remoli M.E., Giacomini E., Severa M., Grillot R., Coccia E.M. (2010). Enhancement of anti-Aspergillus T helper type 1 response by interferon-β-conditioned dendritic cells. Immunology.

[78] Espinosa V., Dutta O., McElrath C., Du P., Chang Y.-J., Cicciarelli B., Pitler A., Whitehead I., Obar J.J., Durbin J.E. (2017). Type III interferon is a critical regulator of innate antifungal immunity. Sci. Immunol..

[79] Wauters E., Van Mol P., Garg A.D., Jansen S., Van Herck Y., Vanderbeke L., Bassez A., Boeckx B., Malengier-Devlies B., Timmerman A. (2021). Discriminating mild from critical COVID-19 by innate and adaptive immune single-cell profiling of bronchoalveolar lavages. Cell Res..

[80] Kyrmizi I., Gresnigt M.S., Akoumianaki T., Samonis G., Sidiropoulos P., Boumpas D., Netea M., Van de Veerdonk F.L., Kontoyiannis D.P., Chamilos G. (2013). Corticosteroids block autophagy protein recruitment in Aspergillus fumigatus phagosomes via targeting Dectin-1/syk kinase signaling. J. Immunol..

[81] Korn T., Mitsdoerffer M., Croxford A.L., Awasthi A., Dardalhon V.A., Galileos G., Vollmar P., Stritesky G.L., Kaplan M.H., Waisman A. (2008). IL-6 controls Th17 immunity in vivo by inhibiting the conversion of conventional T cells into Foxp3+ regulatory T cells. Proc. Natl. Acad. Sci. USA.

[82] Akoumianaki T., Vaporidi K., Diamantaki E., Pène F., Beau R., Gresnigt M., Gkountzinopulou M., Venichaki M., Drakos E., El-Benna J. (2021). Uncoupling of IL-6 signaling and LC3-associated phagocytosis drives immunoparalysis during sepsis. Cell Host Microbe.

[83] Simpson S., Kay F.U., Abbara S., Bhalla S., Chung J.H., Chung M., Henry T.S., Kanne J.P., Kligerman S., Ko J.P. (2020). Radiological Society of North America Expert Consensus Statement on Reporting Chest CT Findings Related to COVID-19. Endorsed by the Society of Thoracic Radiology, the American College of Radiology, and RSNA—Secondary Publication. J. Thorac. Imaging.

[84] Franquet T., Müller N.L., Giménez A., Guembe P., De La Torre J., Bagué S. (2001). Spectrum of pulmonary aspergillosis: Histologic, clinical, and radiologic findings. Radiographics.

[85] Herbrecht R., Guffroy B., Danion F., Venkatasamy A., Simand C., Ledoux M.-P. (2020). Validation by Real-life Data of the New Radiological Criteria of the Revised and Updated Consensus Definition for Invasive Fungal Diseases. Clin. Infect. Dis..

[86] Li Y., Xia L. (2020). Coronavirus disease 2019 (COVID-19): Role of chest CT in diagnosis and management. Am. J. Roentgenol..

[87] Mondoni M., Papa G.F.S., Rinaldo R., Faverio P., Marruchella A., D’Arcangelo F., Pesci A., Pasini S., Henchi S., Cipolla G. (2020). Utility and safety of bronchoscopy during the SARS-CoV-2 outbreak in Italy: A retrospective, multicentre study. Eur. Respir. J..

[88] Chang S.H., Jiang J., Kon Z.N., Williams D.M., Geraci T.C., Smith D.E., Cerfolio R.J., Zervos M., Bizekis C. (2021). Safety and Efficacy of Bronchoscopy in Critically Ill Patients with Coronavirus Disease 2019. Chest.

[89] Levra S., Veljkovic A., Comune M., Bernardi V., Sandri A., Indellicati D. (2021). Bronchoscopy in times of COVID-19 pandemic: An interventional pulmonology unit experience. Respir. Med. Res..

[90] Gao C.A., Bailey J.I., Walter J.M., Coleman J.M., Malsin E.S., Argento A.C., Prickett M.H., Wunderink R.G., NU COVID Investigators (2021). Bronchoscopy on Intubated Patients with COVID-19 is Associated with Low Infectious Risk to Operators. Ann. Am. Thorac. Soc..

[91] Torrego A., Pajares V., Fernández-Arias C., Vera P., Mancebo J. (2020). Bronchoscopy in Patients with COVID-19 with Invasive Mechanical Ventilation: A Single-Center Experience. Am. J. Respir. Crit. Care Med..

[92] Wahidi M.M., Lamb C., Murgu S., Musani A., Shojaee S., Sachdeva A., Maldonado F., Mahmood K., Kinsey M., Sethi S. (2020). American Association for Bronchology and Interventional Pulmonology (AABIP) Statement on the Use of Bronchoscopy and Respiratory Specimen Collection in Patients with Suspected or Confirmed COVID-19 Infection. J. Bronchol. Interv. Pulmonol..

[93] Roman-Montes A.C.M., Martinez-Gamboa A., Diaz-Lomelí P., Cervantes-Sanchez A., Rangel-Cordero A., Sifuentes-Osornio J., Ponce-de-Leon A., Lara M.F.G. (2021). Accuracy of galactomannan testing on tracheal aspirates in COVID-19-associated pulmonary aspergillosis. Mycoses.

[94] Mercier T., Castagnola E., Marr K.A., Wheat L.J., Verweij P.E., Maertens J.A. (2021). Defining Galactomannan Positivity in the Updated EORTC/MSGERC Consensus Definitions of Invasive Fungal Diseases. Clin. Infect. Dis..

[95] Mercier T., Dunbar A., Veldhuizen V., Holtappels M., Schauwvlieghe A., Maertens J., Rijnders B., Wauters J. (2020). Point of care aspergillus testing in intensive care patients. Crit. Care.

[96] Autier B., Prattes J., White P.L., Valerio M., Machado M., Price J., Egger M., Gangneux J.-P., Hoenigl M. (2021). Aspergillus Lateral Flow Assay with Digital Reader for the Diagnosis of COVID-19 Associated Pulmonary Aspergillosis (CAPA): A multicenter study. J. Clin. Microbiol..

[97] Rijnders B.J.A., Schauwvlieghe A.F.A.D., Wauters J. (2020). Influenza-Associated Pulmonary Aspergillosis: A Local or Global Lethal Combination?. Clin. Infect. Dis..

[98] Coste A., Frérou A., Raute A., Couturaud F., Morin J., Egreteau P.-Y., Blanc F.-X., Reignier J., Tadié J.-M., Tran A. (2021). The Extent of Aspergillosis in Critically Ill Patients with Severe Influenza Pneumonia. Crit. Care Med..

[99] Martin-Loeches I., Schultz M.J., Vincent J.L., Alvarez-Lerma F., Bos L.D., Solé-Violán J., Torres A., Rodriguez A. (2017). Increased Incidence of Co-Infection in Critically Ill Patients with Influenza. Intensive Care Med..

[100] Pastores S.M., Dulu A., Voigt L., Raoof N., Alicea M., Halpern N.A. (2007). Premortem clinical diagnoses and postmortem autopsy findings: Discrepancies in critically ill cancer patients. Crit. Care.

[101] Jia S., Gao K., Huang P., Guo R., Zuo X., Xia Q., Hu S., Yu Z., Xie Y. (2021). Interactive Effects of Glucocorticoids and Cytochrome P450 Polymorphisms on the Plasma Trough Concentrations of Voriconazole. Front. Pharm..

[102] Van Daele R., Bekkers B., Lindfors M., Broman L.M., Schauwvlieghe A., Rijnders B., Hunfeld N.G.M., Juffermans N.P., Taccone F.S., Sousa C.A.C. (2021). A large retrospective assessment of voriconazole exposure in patients treated with extracorporeal membrane oxygenation. Microorganisms.

[103] Li M.X., Zhu L.Q., Chen L., Li N., Qi F. (2018). Assessment of drug–drug interactions between voriconazole and glucocorticoids. J. Chemother..

[104] Gautier-Veyret E., Truffot A., Bailly S., Fonrose X., Thiebaut-Bertrand A., Tonini J., Cahn J., Stanke-Labesque F. (2018). Inflammation is a potential risk factor of voriconazole overdose in hematological patients. Fundam. Clin. Pharm..

[105] Gautier-Veyret E., Thiebaut-Bertrand A., Roustit M., Bolcato L., Depeisses J., Schacherer M., Schummer G., Fonrose X., Stanke-Labesque F. (2021). Optimization of voriconazole therapy for treatment of invasive aspergillosis: Pharmacogenomics and inflammatory status need to be evaluated. Br. J. Clin. Pharm..

[106] Ventura M.A.E., Van Wanrooy M.J.P., Span L.F.R., Rodgers M.G.G., Van Den Heuvel E.R., Uges D.R.A., Van Der Werf T.S., Kosterink J.G.W., Alffenaar J.W.C. (2016). Longitudinal analysis of the effect of inflammation on voriconazole trough concentrations. Antimicrob. Agents Chemother..

[107] Maertens J., Selleslag D., Heinz W.J., Saulay M., Rahav G., Giladi M., Aoun M., Kovanda L., Kaufhold A., Engelhardt M. (2018). Treatment outcomes in patients with proven/probable vs possible invasive mould disease in a phase III trial comparing isavuconazole vs voriconazole. Mycoses.

[108] Kaindl T., Andes D., Engelhardt M., Saulay M., Larger P., Groll A.H. (2019). Variability and exposure-response relationships of isavuconazole plasma concentrations in the Phase 3 SECURE trial of patients with invasive mould diseases. J. Antimicrob. Chemother..

[109] Maertens J.A., Rahav G., Lee D.G., Ponce-de-León A., Ramírez Sánchez I.C., Klimko N., Sonet A., Haider S., Diego Vélez J., Raad I. (2021). Posaconazole versus voriconazole for primary treatment of invasive aspergillosis: A phase 3, randomised, controlled, non-inferiority trial. Lancet.

[110] Patterson T.F., Thompson G.R., Denning D.W., Fishman J.A., Hadley S., Herbrecht R., Kontoyiannis D.P., Marr K.A., Morrison V.A., Nguyen M.H. (2016). Practice Guidelines for the Diagnosis and Management of Aspergillosis: 2016 Update by the Infectious Diseases Society of America. Clin. Infect. Dis..

[111] Hoenigl M., Sprute R., Egger M., Arastehfar A., Cornely O.A., Krause R., Lass-Flörl C., Prattes J., Spec A., Thompson G.R. (2021). The Antifungal Pipeline: Fosmanogepix, Ibrexafungerp, Olorofim, Opelconazole, and Rezafungin. Drugs.

[112] Liesenborghs L., Spriet I., Jochmans D., Belmans A., Gyselinck I., Teuwen L.-A., ter Horst S., Dreesen E., Geukens T., Engelen M.M. (2021). Itraconazole for COVID-19: Preclinical studies and a proof-of-concept randomized clinical trial. EBioMedicine.

[113] Conte J.E., Golden J.A., Kipps J., McIver M., Zurlinden E. (2004). Intrapulmonary pharmacokinetics and pharmacodynamics of itraconazole and 14-hydroxyitraconazole at steady state. Antimicrob. Agents Chemother..

[114] Groves H.E., Piché-Renaud P.-P., Peci A., Farrar D.S., Buckrell S., Bancej C., Sevenhuysen C., Campigotto A., Gubbay J.B., Morris S.K. (2021). The impact of the COVID-19 pandemic on influenza, respiratory syncytial virus, and other seasonal respiratory virus circulation in Canada: A population-based study. Lancet Reg. Health Am..

[115] Schwartz I.S., Friedman D.Z.P., Zapernick L., Dingle T.C., Lee N., Sligl W., Zelyas N., Smith S.W. (2020). High Rates of Influenza-Associated Invasive Pulmonary Aspergillosis May Not Be Universal: A Retrospective Cohort Study from Alberta, Canada. Clin. Infect. Dis..

[116] Feys S.R.L., Shoham S., Rijnders B.J.A., Wauters J. (2021). Influenza-Associated Pulmonary Aspergillosis: Seek, and You Shall Find!. Crit. Care Med..

[117] Wauters J., Baar I., Meersseman P., Meersseman W., Dams K., De Paep R., Lagrou K., Wilmer A., Jorens P., Hermans G. (2012). Invasive pulmonary aspergillosis is a frequent complication of critically ill H1N1 patients: A retrospective study. Intensive Care Med..

[118] Van De Veerdonk F.L., Kolwijck E., Lestrade P.P.A., Hodiamont C.J., Rijnders B.J.A., Van Paassen J., Haas P.J., Dos Santos C.O., Kampinga G.A., Bergmans D.C.J.J. (2017). Influenza-associated aspergillosis in critically ill patients. Am. J. Respir. Crit. Care Med..

[119] Vanderbeke L., Janssen N.A.F., Bergmans D.C.J.J., Bourgeois M., Buil J.B., Debaveye Y., Depuydt P., Feys S., Hermans G., Hoiting O. (2021). Posaconazole for prevention of invasive pulmonary aspergillosis in critically ill influenza patients (POSA-FLU): A randomised, open-label, proof-of-concept trial. Intensive Care Med..

