# Supplementary material for: A Visual and Comprehensive Review on COVID-19-Associated Pulmonary Aspergillosis (CAPA)
Source: J Fungi (Basel). 2021 Dec 11;7(12):1067. doi: 10.3390/jof7121067 (PMC8708864; doi:10.3390/jof7121067)
Supplement: Supplementary file 1 [file jof-07-01067-s001.zip › jof-1499953-supplementary.pdf]

Table S1. General information on observational studies and case series with 5 or more CAPA cases publicly available on October 12th 2021.

Study	Study design	Date publication	Country or countries	Setting	Diagnostic criteria	Included patients (n)	Total proven CAPA (n)	Total probable or putative CAPA (n)	Total possible CAPA (n)	Total number of patients with colonization (n)	Percentage proven, probable or putative CAPA among all included patients
Gangneux et al [1]	Prospective observational	Preprint	France	ICU	ECMM/ISHAM allowing TA, AspICU	509		76	24		19.6%
Hatzl et al [2]	Prospective interventional	15-09-2021	Austria	ICU	ECMM/ISHAM	57[1]		8	1		14.0%
Janssen et al [3]	Prospective / retrospective observational	14-09-2021	Belgium, The Netherlands, France	ICU	ECMM/ISHAM	823	6	53	4		7.2%
Ergün et al [4]	Prospective observational	8-09-2021	The Netherlands, Belgium, France, United Kingdom	ICU	ECMM/ISHAM	219	1	38	19	21	17.8%
Prattes et al [5]	Prospective observational	26-08-2021	Austria, Belgium, France, Germany, Italy, Pakistan, Spain, United Kingdom, United States	ICU	ECMM/ISHAM	592	11	80	18		15.4%
Van Ackerbroeck et al [6]	Retrospective observational	19-08-2021	Belgium	ICU	ECMM/ISHAM	18[1]		11			61.1%
Szabo et al [7]	Retrospective observational case series	14-08-2021	Hungary	ICU	EORTC/MSGERC, ECMM/ISHAM & modified AspICU	90		16			17.8%
Iqbal et al [8]	Prospective observational	7-08-2021	Pakistan	ICU	ECMM/ISHAM allowing TA culture	307		61			19.9%
Paramythiotou et al [9]	Retrospective observational	14-07-2021	Greece	ICU	ECMM/ISHAM	179		4	2		2.2%

Vélez et al [10]	Retrospective observational	14-07-2021	Mexico	ICU	ECMM/ISHAM	83	2	14			19.3%
Fortarezza et al [11]	Prospective observational, autopsies	4-07-2021	Italy	HOS	ECMM/ISHAM	45	9				20.0%
Kula et al [12]	Systematic review, autopsies	23-06-2021	Worldwide	ICU	EORTC/MSGERC	677	8				1.2%
Wasylyshyn et al [13]	Retrospective observational	18-06-2021	USA	ICU	EORTC/MSGERC, ECMM/ISHAM	256		2	1		0.8%
Ghazanfari et al [14]	Prospective observational	14-06-2021	Iran	ICU	EORTC/MSGERC, AspICU, ECMM/ISHAM	105[2]		22			21.0%
Oliva et al [15]	Retrospective observational	26-05-2021	Italy	ICU	Modified AspICU	55		2			3.6%
Reizine et al [16]	Retrospective observational	15-05-2021	France	ICU	AspICU, IAPA expert and EORTC/MSGERC allowing NBL	49		4	6		8.2%
Martín et al [17]	Retrospective observational	20-04-2021	Spain	ICU	AspICU	15		3			20.0%
Ripa et al [18]	Prospective observational	27-03-2021	Italy	ICU[3]	AspICU allowing BAL GM	86[3]		10			11.6%
Lahmer et al [19]	Prospective observational	17-03-2021	Germany	ICU	Modified AspICU allowing NBL	32		11		3	34.4%
Permpalung et al [20]	Retrospective observational	9-03-2021	USA	ICU	own expanded CAPA definitions	396		20	19		5.1%
Van Grootveld et al [21]	Prospective observational	5-03-2021	The Netherlands	ICU	ECMM/ISHAM	63		11		2	17.5%
Yusuf et al [22]	Case-control / retrospective observational	18-02-2021	The Netherlands	ICU	No criteria used	92					0.0%
Versyck et al [23]	Retrospective observational	16-02-2021	France	ICU	Modified AspICU	54		2			3.7%
Meijer et al [24]	Prospective case series / observational	16-02-2021	The Netherlands	ICU	ECMM/ISHAM	66		8	5		12.1%
Fekkar et al [25]	Retrospective observational	1-02-2021	France	ICU	EORTC/MSGERC or own criteria (putative)	145		6		25	4.1%
Maes et al [26]	Retrospective observational	11-01-2021	United Kingdom	ICU	Modified AspICU	81		3			3.7%

Marr et al [27]	Retrospective case series	1-01-2021	USA, Spain	HOS	Own criteria	20		20			100.%
Razazi et al [28]	Retrospective observational	18-12-2020	France	ICU	AspICU, IAPA expert and modified AspICU	90		13		4	14.4%
Dellière et al [29]	Retrospective observational	13-12-2020	France	ICU	EORTC/MSGERC, IAPA expert	366		21			5.7%
Benedetti et al [30]	Retrospective case series	7-12-2020	Argentina	ICU	AspICU, IAPA and EORTC/MSGERC	5		5			100.%
Nebreda-Mayoral et al [31]	Retrospective observational	3-12-2020	Spain	HOS	Criteria by Armstrong-James et al	113		3			2.7%
Segrelles-Calvo et al [32]	Prospective observational	3-12-2020	Spain	ICU	EORTC/MSGERC	215		7			3.3%
Machado et al [33]	Prospective observational	29-11-2020	Spain	ICU	Modified AspICU, EORTC/MSGERC	239		8		9	3.3%
Mitaka et al [34]	Retrospective case series	1-10-2020	USA	ICU	AspICU	7		4		3	57.1%
White et al [35]	Prospective observational	29-09-2020	United Kingdom	ICU	AspICU, modified AspICU and own CAPA definitions, allowing NBL	135		20[6]			14.8%
Dupont et al [36]	Prospective observational	10-09-2020	France	ICU	AspICU	106		19			17.9%
Sarrazyn et al [37]	Retrospective observational	9-09-2020	Belgium	HOS	Criteria by Alanio et al	131[7]		4			3.1%
Falces-Romero et al [38]	Retrospective case series	4-08-2020	Spain	HOS	AspICU, EORTC/MSGERC	10		8		2	80.0%
Bartoletti et al [39]	Prospective observational	28-07-2020	Italy	ICU	IAPA criteria	108[8]	2	28		1	27.8%
Nasir et al [40]	Retrospective observational	18-07-2020	Pakistan	ICU	Authors own criteria	23		5		4	21.7%
Van Biesen, et al [41]	Prospective observational	15-07-2020	The Netherlands	ICU	AspICU without host factor, allowing NBL GM	42		9		1	21.4%
Gangneux et al [42]	Prospective observational	10-07-2020	France	ICU	AspICU, authors own modified AspICU	45		9			20.0%
Lamoth et al [43]	Retrospective observational	10-07-2020	Switzerland	ICU	IAPA criteria allowing consecutive bronchial aspirates	118		3			2.5%

Van Arkel et al [44]	Retrospective observational	1-07-2020	The Netherlands	ICU	IAPA criteria, EORTC/MSGERC	31		3	3		9.7%
Wang et al [45]	Retrospective case series / observational	5-06-2020	China	ICU[1]	EORTC/MSGERC	26[1]		8			30.8%
Rutsaert et al [46]	Retrospective case series / observational	1-06-2020	Belgium	ICU	AspICU, EORTC/MSGERC	34	4				11.8%
Alanio et al [47]	Prospective observational	20-05-2020	France	ICU	EORTC/MSGERC, extended version of modified AspICU	27		8			29.6%
Koehler et al [48]	Retrospective chart review	15-04-2020	Germany	ICU	Extended modified AspICU, EORTC/MSGERC	19		5			26.3%
**Observational studies (n=42)**						**6193**	**35**	**638**	**102**	**70**	**10.9%**
**ICU-only observational studies (n=39)**						**5904**	**26**	**631**	**102**	**70**	**11.1%**
**ICU-only retrospective (or partially prospective) observational studies[9] (n=21)**						**2951**	**12**	**198**	**35**	**33**	**7.1%**
**ICU-only prospective observational studies[9] (n=18)**						**2953**	**14**	**433**	**67**	**37**	**15.1%**

Abbreviations: CAPA: COVID-19-associated pulmonary aspergillosis; ECMM/ISHAM: European Confederation of Medical Mycology, International Society for Human & Animal Mycology; EORTC/MSGERC: European Organisation for Research and Treatment of Cancer Studies and Mycosis Study Group Education and Research Consortium; GM: galactomannan; IAPA: influenza-associated pulmonary aspergillosis; NBL: non-bronchoscopic lavage; TA: tracheal aspirate

Remarks: Case series that mentioned observational data were counted as observational studies.

[1]Hatzl et al, Van Ackerbroeck and Wang et al et al: only patients who did not receive prophylaxis are included in this table. [2]Ghazanfari et al: only patients with mechanical ventilation for 4 days or longer were included. [3]Ripa et al: only ICU-admitted patients are mentioned in this table. [4]Yusuf et al: no CAPA diagnosis was given, but 10 of 92 ICU COVID-19 patients had positive tests for *Aspergillus* on lower respiratory tract samples. [5]Dellière et al: probable CAPA was diagnosed in 108 patients (19.4%) who underwent respiratory sampling for deterioration. [6]White et al: 20 proven/putative CAPA patients is the number according to the IAPA criteria. [7]Sarrazyn et al: unclear how many of the included patients were ICU-admitted patients. [8]Bartoletti et al: total ICU population was 185, only the 108 patients with screening for aspergillosis were included in this study. [9]The partially prospective, partially retrospective trial by Janssen et al is counted as a retrospective study.

Table S2. Mortality numbers among CAPA and non-CAPA patients in non-autopsy observational trials in which this data is available.

Study	Mortality endpoint (days or ICU discharge)	Total number of included patients (n)	CAPA patients (proven, probable, putative, possible) (n)	Number of CAPA patients that died (proven, probable, putative, possible), all-cause mortality (n)	Percentage of CAPA patients that died	Total number of patients without arguments for CAPA*	Total number of patients without arguments for CAPA that died*	Percentage of patients without arguments for CAPA that died*
Gangneux et al [1]	ICU discharge	509	100	58	58.0%	409	128	31.3%
Hatzl et al [2]	30	57[1]	9	3	33.3%	48		
Janssen et al [3]	ICU discharge	823[2]	63	31	49.2%	753	192	25.5%
Ergün et al [4]	30	219	58	29	50.0%	133	32	24.1%
Prattes et al [5]	84	592	109	61	56.0%	483	198	41.0%
Iqbal et al [8]	ICU discharge	307	61	56	91.8%	246		
Paramythiotou et al [9]	ICU discharge	179	6	4	66.7%	173		
Vélez et al [10]	60	83	16	5	31.3%	67	9	13.4%
Wasylyshyn et al [13]	84	256	3	1	33.3%	253	61	24.1%
Ghazanfari et al [14]	ICU discharge	105[3]	22	20	90.9%	65	65	100%
Oliva et al [15]	30	55	2	0	0.0%	53	38	71.7%
Reizine et al [16]	90	49	10	3	30.0%	39	8	20.5%
Martín et al [17]	ICU discharge	15	3	1	33.3%	12	2	16.7%
Lahmer et al [19]	ICU discharge	32	11	4	36.4%	21	2	9.5%
Permpalung et al [20]	ICU discharge	396	39	22	56.4%	357	144	40.3%
Van Grootveld et al [21]	ICU discharge	63	11	7	63.6%	44	9	20.5%
Versyck et al [23]	ICU discharge	54	2	2	100.0%	52		
Meijer et al [24]	ICU discharge	66	13	6	46.2%	53		
Fekkar et al [25]	30	145	6	4	66.7%	106		
Maes et al [26]	ICU discharge	81	3	1	33.3%	78	30	38.5%
Marr et al [27]	Not mentioned	20	20	4	20.0%			
Dellière et al [29]	Not mentioned	366	21	15	71.4%			
Nebreda-Mayoral et al [31]	ICU discharge	113	3	2	66.7%			

Segrelles-Calvo et al [32]	ICU discharge	215	7	6	85.7%	208	77	37.0%
Machado et al [33]	ICU discharge	239	8	8	100%	222		
White et al [35]	30	135	20[4]	5	25.0%	84		
Dupont et al [36]	42	106	19	7	36.8%	87		
Sarrazyn et al [37]	ICU discharge	131	4	4	100%	127		
Bartoletti et al [39]	ICU discharge	108[5]	30	13	43.3%	73		
Nasir et al [40]	35	23	5	3	60.0%	14		
Van Biesen, et al [41]	ICU discharge	42	9	2	22.2%	32		
Gangneux et al [42]	ICU discharge	45	9	2	22.2%	30	4	13.3%
Lamoth et al [43]	ICU discharge	118	3	1	33.3%	115		
Van Arkel et al [44]	ICU discharge	31	6	4	66.7%	25	8	32.0%
Rutsaert et al [46]	ICU discharge	34	4	1	25.0%	27		
Alanio et al [47]	ICU discharge	27	8	4	50.0%	18	7	38.9%
Koehler et al [48]	ICU discharge	19	5	3	60.0%	14		
**Total**		**5620**	**728 / 539***	**402 / 249***	**55.2% / 46.2%***	**4521 / 3237***	**1014***	**31.3%***

Remarks: Case series that mentioned observational data were counted as observational studies. *Numbers for studies that provide clear data on the mortality in patients that did not have arguments for aspergillosis (including no patients explicitly categorized as colonized with *Aspergillus*). [1]Hatzl et al: only patients who did not receive prophylaxis are included in this table. [2]Janssen et al: only mortality data for 817 patients available. [3]Ghazanfari et al: only patients with mechanical ventilation for 4 days or longer were included. [4]White et al: 20 proven/putative CAPA patients is the number according to the IAPA criteria. [5]Bartoletti et al: total ICU population was 185, only the 108 patients with screening for aspergillosis were included in this study.

Table S3. Data on *Aspergillus* culture and galactomannan.

Study	Total number included patients (n)	Number proven, probable or putative CAPA among all included patients	Total number of proven, probable, putative or possible CAPA patients (n)	Total number of patients with serum GM (n)	Total number of patients with positive serum GM (n)	Total number of patients with BAL/NBL GM (n)	Total number of patients with positive BAL/NBL GM (n)	Total number of patients with BAL/NBL culture (n)	Total number of patients with positive BAL/NBL culture (n)	Total number of patients with TA culture (n)	Total number of patients with positive TA culture (n)
Gangneux et al [1]	509	76	100								
Hatzl et al [2]	57[1]	8	9		4		7		4		
Janssen et al [3]	823	59	63	349	7	213	45	301	28		
Ergün et al [4]	219	39	58	188	8	71	22	77	17		
Prattes et al [5]	592	91	109								
Van Ackerbroeck et al [6]	18[1]	11	11								
Szabo et al [7]	90	16	16								
Iqbal et al [8]	307	61	61								61
Paramythiotou et al [9]	179	4	6		4					6	5
Vélez et al [10]	83	16	16		5	At least 35	7		2		
Fortarezza et al [11]	45	9	9								
Kula et al [12]	677	8	8								
Wasylyshyn et al [13]	256	2	3		1		1			3	2
Ghazanfari et al [14]	105[2]	22	22	101	5	105	16				
Oliva et al [15]	55	2	2			39	2	55	0		
Reizine et al [16]	49	4	10		3				9		
Martín et al [17]	15	3	3				3	4	3		
Ripa et al [18]	86[3]	10	10								
Lahmer et al [19]	32	11	11	32	4	32	11		9		
Permpalung et al [20]	396	20	39		8		2		2		
Van Grootveld et al [21]	63	11	11				9		3		

Yusuf et al [22]	92	0[4]	0[4]			20	3	24	2		
Versyck et al [23]	54	2	2	54	2		1		1	54	2
Meijer et al [24]	66	8	13		0		2		8		5
Fekkar et al [25]	145	6	6	145	1	/	4	347 BAL sent to lab	3	120 TA sent to lab	2
Maes et al [26]	81	3	3				2				
Marr et al [27]	20	20	20			16	4		3		14
Razazi et al [28]	90	13	13	88	5	0	0	24	4		
Dellière et al [29]	366	21	21								
Benedetti et al [30]	5	5	5	5	5					5	3
Nebreda-Mayoral et al [31]	113	3	3						3		
Segrelles-Calvo et al [32]	215	7	7						6		2
Machado et al [33]	239	8	8		4				7		1
Mitaka et al [34]	7	4	4		1			4	4		
White et al [35]	135	20[5]	20[5]		2	135	17		8		
Dupont et al [36]	106	19	19		1		6		13		2
Sarrazyn et al [37]	131	4	4								4
Falces-Romero et al [38]	10	8	8	2	1	2	2	1	1	8	8
Bartoletti et al [39]	108[6]	30	30	59	1	108	30	189 BAL sent to lab	19		
Nasir et al [40]	23	5	5	23	0						
Van Biesen, et al [41]	42	9	9			42	9	42	7		
Gangneux et al [42]	45	9	9	32	3			45	9		
Lamoth et al [43]	118	3	3	118	1					80	3
Van Arkel et al [44]	31	3	6	3	0		3		2		2
Wang et al [45]	26[1]	8	8								
Rutsaert et al [46]	34	4	4		1		6				1
Alanio et al [47]	27	8	8		1		2	20	7	7	2
Koehler et al [48]	19	5	5		2		3		1		2

Abbreviations: BAL: bronchoalveolar lavage fluid; CAPA: COVID-19-associated pulmonary aspergillosis; GM: galactomannan; IAPA: influenza-associated pulmonary aspergillosis; NBL: non-bronchoscopic lavage fluid; TA: tracheal aspirate

Remarks: If information was not found, cell is left open. [1]Hatzl et al, Van Ackerbroeck et al and Wang et al: only patients who did not receive prophylaxis are included in this table. [2]Ghazanfari et al: only patients with mechanical ventilation for 4 days or longer were included. [3]Ripa et al: only ICU-admitted patients are mentioned in this table. [4]Yusuf et al: no CAPA diagnosis was given, but 10 of 92 ICU COVID-19 patients had positive tests for *Aspergillus* on lower respiratory tract samples. [5]White et al: 20 proven/putative CAPA patients is the number according to the IAPA criteria. [6]Bartoletti et al: total ICU population was 185, only the 108 patients with screening for aspergillosis were included in this study.

Table S4. Cultured *Aspergillus* species and antifungal treatment.

Study	Total number included patients (n)	Number proven, probable or putative CAPA among all included patients	Total number of proven, probable, putative, possible CAPA patients (n)	*Aspergillus* species	Antifungal therapy given to the CAPA cases
Gangneux et al [1]	509	76	100		44 patients VRC, 20 patients L-AMB, 16 patients caspofungin, 11 patients ISV and 3 patients other antifungal drugs
Hatzl et al [2]	57[1]	8	9		3 patients ISV, 6 POS, 1 POS then ISV
Janssen et al [3]	823	59	63	*A. fumigatus* in the majority of patients	Discovery cohort: 20 patients VRC, 13 patients azole + echinocandin combination therapy, 2 patients other azole monotherapy, 1 patient echinocandin monotherapy, 1 patient L-AMB monotherapy
Ergün et al [4]	219	39	58		19 patients VRC, 28 patients VRC/Anidulafungin, 9 patients L-AMB, 2 patients Caspofungin, 1 patient VRC/Caspofungin, 1 patient VRC/L-AMB
Prattes et al [5]	592	91	109		
Van Ackerbroeck et al [6]	18	11	11		
Szabo et al [7]	90	16	16	*A.fumigatus* in all patients	
Iqbal et al [8]	307	61	61	*A. fumigatus* (78.7%), *A. flavus* (16.4%), *A. terreus* (3.3%) and *A. niger* (1.6%)	59 patients VRC, 2 patients VRC/L-AMB
Paramythiotou et al [9]	179	4	6	*A. fumigatus* (patient 1), *A. flavus* (patient 2), *A. flavus* and *A. fumigatus* (patient 3), *A. fumigatus* and *A. terreus* (patient 4), *A. terreus* (patient 6)	5 patients ISV, 1 patient Caspofungin/L-AMB
Vélez et al [10]	83	16	16		
Fortarezza et al [11]	45	9	9		/
Kula et al [12]	677	8	8		1 patient L-AMB/ISV, many patients undocumented
Wasylyshyn et al [13]	256	2	3	*A.fumigatus* in two patients	1 patient IV VRC (2 weeks) followed by combination of micafungin and L-AMB, 1 patient PO VRC/PO ISV daily
Ghazanfari et al [14]	105[2]	22	22	*A. flavus* (63,6%), A. fumigatus and *A. japonicus* (13,6%) and *A. niger* (9,1%)	/
Oliva et al [15]	55	2	2	/	No
Reizine et al [16]	49	4	10		8 patients VRC

Martín et al [17]	15	3	3	*A. fumigatus*	
Ripa et al [18]	86[3]	10	10		
Lahmer et al [19]	32	11	11	*A. fumigatus* (81.8%) in all putative cases	5 patients VRC, 1 patient ISV and 5 patients L-AMB
Permpalung et al [20]	396	20	39		
Van Grootveld et al [21]	63	11	11	*A. fumigatus* (92%), *A. niger* (4%) *en A. flavus* (4%)	6 patients treated with antifungals
Yusuf et al [22]	92	0[4]	0[4]		
Versyck et al [23]	54	2	2	*A. fumigatus*	2/2 patients VRC
Meijer et al [24]	66	8	13	*A. fumigatus*	7 patients VRC, 5 patients empiric caspofungin/ VRC/L-AMB, 1 patient L-AMB/ VRC
Fekkar et al [25]	145	6	6	*A. fumigatus*	3 patient VRC and Caspofungin, 1 patient L-AMB and Caspofungin, 1 patient VRC/ L-AMB/caspofungin/ISV, 1 patient VRC/ caspofungin/L-AMB
Maes et al [26]	81	3	3		3 patients L-AMB
Marr et al [27]	20	20	20	*A. fumigatus* (71%)	All but 2 patients were treated with antifungals including: VRC, POS, or L-AMB
Razazi et al [28]	90	13	13		
Dellière et al [29]	366	21	21		
Benedetti et al [30]	5	5	5	*A. fumigatus* (60%)	4 patients VRC, 1 patient L-AMB
Nebreda-Mayoral et al [31]	113	3	3	*A. fumigatus* (66%) and *A. niger* (33%)	
Segrelles-Calvo et al [32]	215	7	7	*A. fumigatus* (43%), *A. flavus* and *A. niger* (28%)	3 patients Itraconazole, 1 patient L-AMB
Machado et al [33]	239	8	8	*A. fumigatus* (75%) *A. citrinoterreus* and *A. lentulus* (12,5%)	4 patients ISV, 1 patient L-AMB
Mitaka et al [34]	7	4	4	*A. fumigatus*	3 patients VRC, 1 patient Caspofungin
White et al [35]	135	20[5]	20[5]	*A. fumigatus*	4 patients VRC, 1 patient L-AMB, 1 patient VRC/L-AMB, Anidulafungin/L-AMB
Dupont et al [36]	106	19	19	*A. fumigatus* (87,5%) and *A. niger* and *A. calidoustus* (6,25%)	8 patients VRC, 1 patient VRC + Caspofungin
Sarrazyn et al [37]	131	4	4		1 patient VRC and 2 patients VRC followed by L-AMB
Falces-Romero et al [38]	10	8	8	*A. fumigatus (*90%) and *A. nidulans* (10%)	2 patients VRC, 1 patient VRC/Caspofungin, 1 patient L-AMB, 1 patient L-AMB/ISV, 1 patient L-AMB/VRC, 1 patient Micafungin/VRC/ISV/L-Amn, 1 patient L-AMB/Anidulafungin
Bartoletti et al [39]	108[6]	30	30	*A. fumigatus* (79%) and *A.niger (*15,8%) and *A. flavus* (5,2%)	13 patients VRC, 3 patients other antifungals

Nasir et al [40]	23	5	5	*A. flavus* (54,5%), *A. niger* (27,3%) and *A. fumigatus* (18,2%)	3 patients VRC, 2 patients L-AMB
Van Biesen, et al [41]	42	9	9	*A. fumigatus* (71%) and *A. flavus* and *A. terreus* (14,3%)	9 patients VRC/L-AMB
Gangneux et al [42]	45	9	9	*A. fumigatus* (88,9%) and *A. niger* (11,1%)	7 patients VRC or ISV
Lamoth et al [43]	118	3	3	*A. fumigatus*	3/3 patients VRC
Van Arkel et al [44]	31	3	6	*A. fumigatus*	5 patients VRC/Anidulafungin and 1 patient L-AMB
Wang et al [45]	26[1]	8	8	*A. fumigatus*	
Rutsaert et al [46]	34	4	4	*A. fumigatus* (83.3%) and *A. flavus* (16,7%)	3 patients VRC, 1 patient VRC/ISV
Alanio et al [47]	27	8	8	*A. fumigatus*	1 patient VRC, 1 patient Caspofungin and 7 other (putative) patients no antifungals
Koehler et al [48]	19	5	5	*A. fumigatus*	2 patients VRC, 2 patients Caspofungin/VRC, 1 patient ISV

Abbreviations: VRC = voriconazole; ISV = isavuconazole; POS = posaconazole; L-AMB = liposomal amphotericin B

Remarks: If information was not found, cell is left open. [1]Hatzl et al, Van Ackerbroeck et al and Wang et al: only patients who did not receive prophylaxis are included in this table. [2]Ghazanfari et al: only patients with mechanical ventilation for 4 days or longer were included. [3]Ripa et al: only ICU-admitted patients are mentioned in this table. [4]Yusuf et al: no CAPA diagnosis was given, but 10 of 92 ICU COVID-19 patients had positive tests for *Aspergillus* on lower respiratory tract samples. [5]White et al: 20 proven/putative CAPA patients is the number according to the IAPA criteria. [6]Bartoletti et al: total ICU population was 185, only the 108 patients with screening for aspergillosis were included in this study.

References

1. Gangneux, J.P.; Dannaoui, E.; Fekkar, A.; Luyt, C.E.; Botterel, F.; de Prost, N.; Tadie, J.M.; Reizine, F.; Houze, S.; Timsit, J.-F.; et al. High Prevalence of Fungal Infections in Mechanically Ventilated COVID-19 Patients in the ICU: The French Multicenter MYCOVID Study. *SSRN Electron. J.* **2021**, doi:10.2139/ssrn.3858565.

2. Hatzl, S.; Reisinger, A.C.; Posch, F.; Prattes, J.; Stradner, M.; Pilz, S.; Eller, P.; Schoerghuber, M.; Toller, W.; Gorkiewicz, G.; et al. Antifungal prophylaxis for prevention of COVID-19-associated pulmonary aspergillosis in critically ill patients: an observational study. *Crit. Care* **2021**, *25*, doi:10.1186/s13054-021-03753-9.

3. Janssen, N.A.F.; Nyga, R.; Vanderbeke, L.; Jacobs, C.; Ergün, M.; Buil, J.B.; Dijk, K. van; Altenburg, J.; Bouman, C.S.C.; Spoel, H.I. van der; et al. Multinational Observational Cohort Study of COVID-19–Associated Pulmonary Aspergillosis - Volume 27, Number 11—November 2021 - Emerging Infectious Diseases journal - CDC. *Emerg. Infect. Dis.* **2021**, *27*, doi:10.3201/EID2711.211174.

4. Ergün, M.; Brüggemann, R.J.M.; Alanio, A.; Dellière, S.; van Arkel, A.; Bentvelsen, R.G.; Rijpstra, T.; van der Sar-van der Brugge, S.; Lagrou, K.; Janssen, N.A.F.; et al. Aspergillus test profiles and mortality in critically-ill COVID-19 patients. *J. Clin. Microbiol.* **2021**, doi:10.1128/jcm.01229-21.

5. Prattes, J.; Wauters, J.; Giacobbe, D.R.; Salmanton-García, J.; Maertens, J.; Bourgeois, M.; Reynders, M.; Rutsaert, L.; Van Regenmortel, N.; Lormans, P.; et al. Risk factors and outcome of pulmonary aspergillosis in critically ill coronavirus disease 2019 patients– a multinational observational study by the European Confederation of Medical Mycology. *Clin. Microbiol. Infect.* **2021**, doi:10.1016/j.cmi.2021.08.014.

6. Van Ackerbroeck, S.; Rutsaert, L.; Roelant, E.; Dillen, K.; Wauters, J.; Van Regenmortel, N. Inhaled liposomal amphotericin-B as a prophylactic treatment for COVID-19-associated pulmonary aspergillosis/aspergillus tracheobronchitis. *Crit. Care* 2021, *25*.

7. Szabo, B.G.; Lakatos, B.; Bobek, I.; Szabo, E.; Szlavik, J.; Vályi-Nagy, I. Invasive fungal infections among critically ill adult COVID-19 patients: First experiences from the national centre in Hungary. *J. Med. Mycol.* **2021**, *31*, doi:10.1016/j.mycmed.2021.101198.

8. Iqbal, A.; Ramzan, M.; Akhtar, A.; Ahtesham, A.; Aslam, S.; Khalid, J. COVID-Associated Pulmonary Aspergillosis and Its Related Outcomes: A Single-Center Prospective Observational Study. *Cureus* **2021**, *13*, doi:10.7759/cureus.16982.

9. Paramythiotou, E.; Dimopoulos, G.; Koliakos, N.; Siopi, M.; Vourli, S.; Pournaras, S.; Meletiadis, J. Epidemiology and Incidence of COVID-19-Associated Pulmonary Aspergillosis (CAPA) in a Greek Tertiary Care Academic Reference Hospital. *Infect. Dis. Ther.* **2021**, *10*, 1779–1792, doi:10.1007/s40121-021-00486-8.

10. Vélez Pintado, M.; Camiro-Zúñiga, A.; Aguilar Soto, M.; Cuenca, D.; Mercado, M.; Crabtree-Ramirez, B. COVID-19-associated invasive pulmonary aspergillosis in a tertiary care center in Mexico City. *Med. Mycol.* **2021**, *59*, 828–833, doi:10.1093/mmy/myab009.

11. Fortarezza, F.; Boscolo, A.; Pezzuto, F.; Lunardi, F.; Acosta, M.J.; Giraudo, C.; Vecchio, C. Del; Sella, N.; Tiberio, I.; Godi, I.; et al. Proven COVID-19—associated pulmonary aspergillosis in patients with severe respiratory failure. *Mycoses* **2021**, *64*, 1223–1229, doi:10.1111/MYC.13342.

12. Kula, B.E.; Clancy, C.J.; Hong Nguyen, M.; Schwartz, I.S. Invasive mould disease in fatal COVID-19: a systematic review of autopsies. *The Lancet Microbe* 2021, *2*, e405–e414.

13. Wasylyshyn, A.I.; Wasylyshyn, G.R.; Linder, K.A.; Miceli, M.H. COVID-19-Associated Pulmonary Aspergillosis at an Academic Medical Center in the Midwestern United States. *Mycopathologia* **2021**, *186*, 499–505, doi:10.1007/s11046-021-00564-y.

14. Ghazanfari, M.; Arastehfar, A.; Davoodi, L.; Yazdani Charati, J.; Moazeni, M.; Abastabar, M.; Haghani, I.; Mirzakhani, R.; Mayahi, S.; Fang, W.; et al. Pervasive but

Neglected: A Perspective on COVID-19-Associated Pulmonary Mold Infections Among Mechanically Ventilated COVID-19 Patients. *Front. Med.* **2021**, *8*, doi:10.3389/fmed.2021.649675.

15. Oliva, A.; Ceccarelli, G.; Borrazzo, C.; Ridolfi, M.; D.'Ettorre, G.; Alessandri, F.; Ruberto, F.; Pugliese, F.; Raponi, G.M.; Russo, A.; et al. Comparison of clinical features and outcomes in COVID-19 and influenza pneumonia patients requiring intensive care unit admission. *Infection* **2021**, *49*, 965–975, doi:10.1007/s15010-021-01624-7.

16. Reizine, F.; Pinceaux, K.; Lederlin, M.; Autier, B.; Guegan, H.; Gacouin, A.; Luque-Paz, D.; Boglione-Kerrien, C.; Bacle, A.; Le Daré, B.; et al. Influenza-and covid-19-associated pulmonary aspergillosis: Are the pictures different? *J. Fungi* **2021**, *7*, doi:10.3390/jof7050388.

17. Sánchez Martín, C.; Madrid Martínez, E.; González Pellicer, R.; Armero Ibáñez, R.; Martínez González, E.; Llau Pitarch, J. V. Invasive pulmonary aspergillosis in patients with acute respiratory syndrome by COVID-19. *Rev. Esp. Anestesiol. Reanim.* **2021**, doi:10.1016/j.redar.2021.02.012.

18. Ripa, M.; Galli, L.; Poli, A.; Oltolini, C.; Spagnuolo, V.; Mastrangelo, A.; Muccini, C.; Monti, G.; De Luca, G.; Landoni, G.; et al. Secondary infections in patients hospitalized with COVID-19: incidence and predictive factors. *Clin. Microbiol. Infect.* **2021**, *27*, 451–457, doi:10.1016/j.cmi.2020.10.021.

19. Lahmer, T.; Kriescher, S.; Herner, A.; Rothe, K.; Spinner, C.D.; Schneider, J.; Mayer, U.; Neuenhahn, M.; Hoffmann, D.; Geisler, F.; et al. Invasive pulmonary aspergillosis in critically ill patients with severe COVID-19 pneumonia: Results from the prospective AspCOVID-19 study. *PLoS One* **2021**, *16*, doi:10.1371/journal.pone.0238825.

20. Permpalung, N.; Chiang, T.P.-Y.; Massie, A.B.; Zhang, S.X.; Avery, R.K.; Nematollahi, S.; Ostrander, D.; Segev, D.L.; Marr, K.A. Coronavirus Disease 2019–Associated Pulmonary Aspergillosis in Mechanically Ventilated Patients. *Clin. Infect. Dis.* **2021**, doi:10.1093/cid/ciab223.

21. van Grootveld, R.; van Paassen, J.; de Boer, M.G.J.; Claas, E.C.J.; Kuijper, E.J.; van der Beek, M.T. Systematic screening for COVID-19 associated invasive aspergillosis in ICU patients by culture and PCR on tracheal aspirate. *Mycoses* **2021**, *64*, 641–650, doi:10.1111/myc.13259.

22. Yusuf, E.; Vonk, A.; van den Akker, J.P.C.; Bode, L.; Sips, G.J.; Rijnders, B.J.A.; de Steenwinkel, J.; Verkaik, N.J.; Vogel, M.; van der Eerden, M.; et al. Frequency of positive aspergillus tests in COVID-19 patients in comparison to other patients with pulmonary infections admitted to the intensive care unit. *J. Clin. Microbiol.* **2021**, *59*, doi:10.1128/JCM.02278-20.

23. Versyck, M.; Zarrougui, W.; Lambiotte, F.; Elbeki, N.; Saint-Leger, P. Invasive pulmonary aspergillosis in COVID-19 critically ill patients: Results of a French monocentric cohort. *J. Mycol. Med.* **2021**, *31*, 101122, doi:10.1016/J.MYCMED.2021.101122.

24. Meijer, E.F.J.; Dofferhoff, A.S.M.; Hoiting, O.; Meis, J.F. COVID-19–associated pulmonary aspergillosis: a prospective single-center dual case series. *Mycoses* **2021**, *64*, 457–464, doi:10.1111/myc.13254.

25. Fekkar, A.; Lampros, A.; Mayaux, J.; Poignon, C.; Demeret, S.; Constantin, J.M.; Marcelin, A.G.; Monsel, A.; Luyt, C.E.; Blaize, M. Occurrence of invasive pulmonary fungal infections in patients with severe COVID-19 admitted to the ICU. *Am. J. Respir. Crit. Care Med.* **2021**, *203*, 307–317, doi:10.1164/rccm.202009-3400OC.

26. Maes, M.; Higginson, E.; Pereira-Dias, J.; Curran, M.D.; Parmar, S.; Khokhar, F.; Cuchet-Lourenço, D.; Lux, J.; Sharma-Hajela, S.; Ravenhill, B.; et al. Ventilator-associated pneumonia in critically ill patients with COVID-19. *Crit. Care* **2021**, *25*, doi:10.1186/s13054-021-03460-5.

27. Marr, K.A.; Platt, A.; Tornheim, J.A.; Zhang, S.X.; Datta, K.; Cardozo, C.; Garcia-Vidal, C. Aspergillosis complicating severe coronavirus disease. *Emerg. Infect. Dis.* **2021**, *27*, 18–25, doi:10.3201/eid2701.202896.

28. Razazi, K.; Arrestier, R.; Haudebourg, A.F.; Benelli, B.; Carteaux, G.; Decousser, J. -W; Fourati, S.; Woerther, P.L.; Schlemmer, F.; Charles-Nelson, A.; et al. Risks of ventilator-associated pneumonia and invasive pulmonary aspergillosis in patients with viral acute respiratory distress syndrome related or not to Coronavirus 19 disease. *Crit. Care* **2020**, *24*, doi:10.1186/s13054-020-03417-0.

29. Dellière, S.; Dudoignon, E.; Fodil, S.; Voicu, S.; Collet, M.; Oillic, P.A.; Salmona, M.; Dépret, F.; Ghelfenstein-Ferreira, T.; Plaud, B.; et al. Risk factors associated with COVID-19-associated pulmonary aspergillosis in ICU patients: a French multicentric retrospective cohort. *Clin. Microbiol. Infect.* **2021**, *27*, 790.e1-790.e5, doi:10.1016/j.cmi.2020.12.005.

30. Benedetti, M.F.; Alava, K.H.; Sagardia, J.; Cadena, R.C.; Laplume, D.; Capece, P.; Posse, G.; Nusblat, A.D.; Cuestas, M.L. COVID-19 associated pulmonary aspergillosis in ICU patients: Report of five cases from Argentina. *Med. Mycol. Case Rep.* **2021**, *31*, 24–28, doi:10.1016/j.mmcr.2020.11.003.

31. Nebreda-Mayoral, T.; Miguel-Gómez, M.A.; March-Rosselló, G.A.; Puente-Fuertes, L.; Cantón-Benito, E.; Martínez-García, A.M.; Muñoz-Martín, A.B.; Orduña-Domingo, A. Bacterial/fungal infection in hospitalized patients with COVID-19 in a tertiary hospital in the Community of Castilla y León, Spain. *Enferm. Infecc. Microbiol. Clin.* **2021**, doi:10.1016/j.eimc.2020.11.003.

32. Segrelles-Calvo, G.; Araújo, G.R.S.; Llopis-Pastor, E.; Carrillo, J.; Hernández-Hernández, M.; Rey, L.; Rodríguez Melean, N.; Escribano, I.; Antón, E.; Zamarro, C.; et al. Prevalence of opportunistic invasive aspergillosis in COVID-19 patients with severe pneumonia. *Mycoses* **2021**, *64*, 144–151, doi:10.1111/myc.13219.

33. Machado, M.; Valerio, M.; Álvarez-Uría, A.; Olmedo, M.; Veintimilla, C.; Padilla, B.; De la Villa, S.; Guinea, J.; Escribano, P.; Ruiz-Serrano, M.J.; et al. Invasive pulmonary aspergillosis in the COVID-19 era: An expected new entity. *Mycoses* 2021, *64*, 132–143.

34. Mitaka, H.; Perlman, D.C.; Javaid, W.; Salomon, N. Putative invasive pulmonary aspergillosis in critically ill patients with COVID-19: An observational study from New York City. *Mycoses* **2020**, *63*, 1368–1372, doi:10.1111/myc.13185.

35. White, P.L.; Dhillon, R.; Cordey, A.; Hughes, H.; Faggian, F.; Soni, S.; Pandey, M.; Whitaker, H.; May, A.; Morgan, M.; et al. A National Strategy to Diagnose Coronavirus Disease 2019–Associated Invasive Fungal Disease in the Intensive Care Unit. *Clin. Infect. Dis.* **2021**, *73*, e1634–e1644, doi:10.1093/cid/ciaa1298.

36. Dupont, D.; Menotti, J.; Turc, J.; Miossec, C.; Wallet, F.; Richard, J.C.; Argaud, L.; Paulus, S.; Wallon, M.; Ader, F.; et al. Pulmonary aspergillosis in critically ill patients with Coronavirus Disease 2019 (Covid-19). *Med. Mycol.* **2021**, *59*, 110–114, doi:10.1093/mmy/myaa078.

37. Sarrazyn, C.; Dhaese, S.; Demey, B.; Vandecasteele, S.; Reynders, M.; Van Praet, J.T. Incidence, risk factors, timing, and outcome of influenza versus COVID-19-associated putative invasive aspergillosis. *Infect. Control Hosp. Epidemiol.* 2021, *42*, 1149–1150.

38. Falces-Romero, I.; Ruiz-Bastián, M.; Díaz-Pollán, B.; Maseda, E.; García-Rodríguez, J.; Montero-Vega, M.D.; Romero-Gómez, M.P.; García-Bujalance, S.; Cendejas-Bueno, E.; Toro-Rueda, C.; et al. Isolation of Aspergillus spp. in respiratory samples of patients with COVID-19 in a Spanish Tertiary Care Hospital. *Mycoses* **2020**, *63*, 1144–1148, doi:10.1111/myc.13155.

39. Bartoletti, M.; Pascale, R.; Cricca, M.; Rinaldi, M.; Maccaro, A.; Bussini, L.; Fornaro, G.; Tonetti, T.; Pizzilli, G.; Francalanci, E.; et al. Epidemiology of Invasive Pulmonary Aspergillosis Among Intubated Patients With COVID-19: A Prospective Study. *Clin. Infect. Dis.* **2020**, doi:10.1093/cid/ciaa1065.

40. Nasir, N.; Farooqi, J.; Mahmood, S.F.; Jabeen, K. COVID-19-associated pulmonary aspergillosis (CAPA) in patients admitted with severe COVID-19 pneumonia: An observational study from Pakistan. *Mycoses* **2020**, *63*, 766–770, doi:10.1111/myc.13135.

41. Biesen, S. Van; Kwa, D.; Bosman, R.J.; Juffermans, N.P. Detection of Invasive Pulmonary Aspergillosis in COVID-19 with Nondirected BAL. *Am. J. Respir. Crit. Care Med.*

2020, *202*, 1171, doi:10.1164/RCCM.202005-2018LE.

42. Gangneux, J.P.; Reizine, F.; Guegan, H.; Pinceaux, K.; Le Balch, P.; Prat, E.; Pelletier, R.; Belaz, S.; Le Souhaitier, M.; Le Tulzo, Y.; et al. Is the COVID-19 pandemic a good time to include aspergillus molecular detection to categorize aspergillosis in ICU patients? A monocentric experience. *J. Fungi* **2020**, *6*, 1–12, doi:10.3390/jof6030105.

43. Lamoth, F.; Glampedakis, E.; Boillat-Blanco, N.; Oddo, M.; Pagani, J.L. Incidence of invasive pulmonary aspergillosis among critically ill COVID-19 patients. *Clin. Microbiol. Infect.* **2020**, *26*, 1706–1708, doi:10.1016/J.CMI.2020.07.010.

44. van Arkel, A.L.E.; Rijpstra, T.A.; Belderbos, H.N.A.; van Wijngaarden, P.; Verweij, P.E.; Bentvelsen, R.G. COVID-19-associated pulmonary aspergillosis. *Am. J. Respir. Crit. Care Med.* 2020, *202*, 132–135.

45. Wang, J.; Yang, Q.; Zhang, P.; Sheng, J.; Zhou, J.; Qu, T.; Qu, T. Clinical characteristics of invasive pulmonary aspergillosis in patients with COVID-19 in Zhejiang, China: A retrospective case series. *Crit. Care* **2020**, *24*, doi:10.1186/s13054-020-03046-7.

46. Rutsaert, L.; Steinfort, N.; Van Hunsel, T.; Bomans, P.; Naesens, R.; Mertes, H.; Dits, H.; Van Regenmortel, N. COVID-19-associated invasive pulmonary aspergillosis. *Ann. Intensive Care* 2020, *10*.

47. Alanio, A.; Dellière, S.; Fodil, S.; Bretagne, S.; Mégarbane, B. Prevalence of putative invasive pulmonary aspergillosis in critically ill patients with COVID-19. *Lancet Respir. Med.* 2020, *8*, e48–e49.

48. Koehler, P.; Cornely, O.A.; Böttiger, B.W.; Dusse, F.; Eichenauer, D.A.; Fuchs, F.; Hallek, M.; Jung, N.; Klein, F.; Persigehl, T.; et al. COVID-19 associated pulmonary aspergillosis. *Mycoses* **2020**, *63*, 528–534, doi:10.1111/myc.13096.
